# Combinatory Exposure to Urolithin A, Alternariol, and Deoxynivalenol Affects Colon Cancer Metabolism and Epithelial Barrier Integrity *in vitro*

**DOI:** 10.3389/fnut.2022.882222

**Published:** 2022-06-24

**Authors:** Julia Groestlinger, Carina Seidl, Elisabeth Varga, Giorgia Del Favero, Doris Marko

**Affiliations:** ^1^Department of Food Chemistry and Toxicology, Faculty of Chemistry, University of Vienna, Vienna, Austria; ^2^Core Facility Multimodal Imaging, Faculty of Chemistry, University of Vienna, Vienna, Austria

**Keywords:** intestinal health, chronic inflammatory diseases, trichothecenes, dibenzo-α-pyrones, food safety, food contaminants

## Abstract

The human gastrointestinal tract is an important site of nutrient absorption and a crucial barrier against xenobiotics. It regularly faces “chemical cocktails” composed of food constituents, their human and microbial metabolites, and foodborne contaminants, such as mycotoxins. Hence, the colonic epithelium adapts to dietary molecules tuning its immune response, structural integrity, and metabolism to maintain intestinal homeostasis. While gut microbiota metabolites of berry ellagitannins, such as urolithin A (Uro A) might contribute to physiological epithelial barrier integrity, foodborne co-contaminating mycotoxins like alternariol (AOH) and deoxynivalenol (DON) could hamper epithelial function. Hence, we investigated the response of differentiated Caco-2 cells (clone C2BBe1) *in vitro* to the three compounds alone or in binary mixtures. In virtue of the possible interactions of Uro A, AOH, and DON with the aryl hydrocarbon receptor (AhR) pathway, potential effects on phase-I-metabolism enzymes and epithelial structural integrity were taken as endpoints for the evaluation. Finally, Liquid chromatography tandem mass spectrometry measurements elucidated the absorption, secretion, and metabolic capacity of the cells under single and combinatory exposure scenarios. Uro A and AOH as single compounds, and as a binary mixture, were capable to induce CYP1A1/1A2/1B1 enzymes triggered by the AhR pathway. In light of its ribosome inhibiting capacity, the trichothecene suppressed the effects of both dibenzo-α-pyrones. In turn, cellular responsiveness to Uro A and AOH could be sustained when co-exposed to DON-3-sulfate, instead of DON. Colonic epithelial structural integrity was rather maintained after incubation with Uro A and AOH: this was reinforced in the combinatory exposure scenario and disrupted by DON, an effect, opposed in combination. Passage through the cells as well as the metabolism of Uro A and AOH were rather influenced by co-exposure to DON, than by interaction with each other. Therefore, we conclude that although single foodborne bioactive substances individually could either support or disrupt the epithelial structure and metabolic capacity of colon cancer, exposure to chemical mixtures changes the experimental outcome and calls for the need of combinatory investigations for proper risk assessment.

## Introduction

Together with our microbiome, more than a milliard of bacteria hosted by the colon, the colonic epithelium not only participates in the absorption/resorption of food constituents but also in their metabolism (1, 2). Indeed, a crucial role of the colonic epithelium is to serve as barrier against xenobiotics. Besides, it hosts immune responses to support the elimination of toxins and pathogens. High cell turnover and constant renewal of the epithelial structures are necessary to fulfill its physiological roles (3–6). This is of relevance also for pathological conditions, such as colon carcinoma, since chronic diseases are often the result of a disrupted epithelium and are accompanied by a disbalance in metabolism and immune signaling (7, 8). Besides, foodborne xenobiotics and microbial metabolites are frequently reported to impact the intestinal epithelial homeostasis and possibly contribute to the genesis of chronic gastrointestinal tract (GIT)-related diseases (9, 10). Hence, metabolic competence is crucial to support detoxification.

A highly conserved signaling pathway involved in this respect is triggered by the activation of the aryl hydrocarbon receptor (AhR), which supports intestinal metabolic capacities, epithelial barrier function (11, 12), and affects colonic inflammatory processes (13), all crucial for intestinal homeostasis (14). In detail, the AhR pathway target genes are involved in Phase I- and Phase II-metabolism and encode for CYP1A1, CYP1A2, CYP1B1, NQ01, ALDH3A1, and UGT1A, and GSTA1 enzymes (15–18). Moreover, activation of the AhR was reported to participate in the maintenance of the intestinal barrier structure by regulating the cellular distribution and expression of tight junction proteins (14). Furthermore, AhR signaling acts on the immune response *via* multiple interactions with transcription factors known to mediate pro-inflammatory processes, such as the IL-1β-induced NF-κB pathway (19).

**FIGURE 1:**
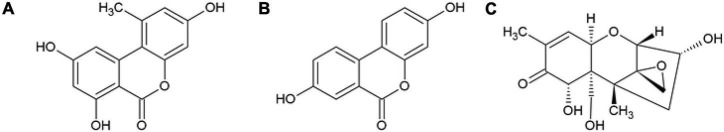
Structures of **(A)** the ellagic acid gut microbiota metabolite, urolithin A (Uro A), **(B)** the *Alternaria alternata* secondary metabolite alternariol (AOH), and **(C)** the *Fusarium* spp. fungal metabolite deoxynivalenol (DON).

A balanced diet offers a great variety of nurturing chemicals contributing to a healthy lifestyle and GIT system. Ellagitannins are commonly occurring constituents of berries, nuts, and fruits, and the extracts of ellagitannin sources are used as dietary supplements (20, 21). Throughout their way through our GIT, ellagitannins are transformed into ellagic acid and further to a group of dibenzo-α-pyrones (DAPs), called urolithins, by the gut microbiome. These microbial metabolites are claimed to be essential for some of the health promoting bioactivities of ellagitannins (22, 23). Protective effects against GIT related diseases, including colorectal cancer, and inflammatory bowel disease were postulated for both, a directly supplemented, as well as *in vivo* generated DAP: urolithin A (Uro A, Figure 1A; 24). Uro A was suggested to restore the colonic epithelial barrier integrity in chronic inflammatory diseases (25). It was reported to activate the Phase I- and Phase II-metabolism driving AhR pathway, acts as an antioxidant *via* the Nrf2-ARE pathway, and exert anti-inflammatory activities (26, 27). Bioavailability of urolithins formed from ellagitannins is frequently reported (28–30). Next to Uro A, human metabolites of Uro A recently recovered in plasma following 4-week oral supplementations included Uro A-glucuronide (Uro A-GlcA) and Uro A-sulfate (Uro A-S; 31).

In addition to food constituents, harmful compounds can also be present in the diet. Fungal secondary metabolites produced by the fungal species, *Alternaria* and *Fusarium* are regularly detected to co-contaminate food and feed products (32, 33). Alternariol (AOH, Figure 1B), a small molecule produced by *Alternaria alternata*, is considered an “emerging mycotoxin”, that requires toxicological risk assessment beyond its reported toxic effects (34, 35). AOH was previously described to hold, amongst others, genotoxic, endocrine disruptive, pro-oxidative, and immunomodulatory potential (36–39). Furthermore, AOH was suggested to impact cell membrane properties relevant for the innate immune response (40). Structurally, AOH belongs to the chemical class of DAPs. Sharing structural similarities with Uro A, it can be hypothesized for both, to exert beneficial as well as detrimental effects to human health, opening the research question concerning their singular and combined mechanisms of action (41). Biomonitoring studies recurringly detect AOH in human urine samples (42, 43). Data on human metabolites *in vivo* are still scarce; however, oxidative metabolites produced in human microsomes include 2-, 4-, 8-, and 10-hydroxy (OH)-AOH (44). A recent animal study also recovered 4-OH-AOH in rat feces after 24 h of oral administration of AOH (45).

*Fusarium* spp. infesting mostly grains, are regularly reported to produce a B-type trichothecene secondary metabolite, deoxynivalenol (DON, Figure 1C; 46). DON, also known as vomitoxin, can cause acute intoxication symptoms affecting the GIT (47). Moreover, gastrointestinal health can be compromised by low chronic exposure to DON as well. In relation to its proteostatic potential, DON was described to modulate the absorption of nutrients (48), and impact on intestinal barrier homeostasis, for instance, by regulating proteins responsible for epithelial structure, leading to disruptions in the epithelial barrier integrity in a time-, and dose-dependent manner (49). Furthermore, DON is known to exert pro-inflammatory as well as immunomodulatory effects *in vitro*, depending on the exposure scenario (48, 50, 51). According to literature, upon ingestion, DON is absorbed, metabolized, and excreted in urine within 24 h. Next to free DON, the recovered human metabolites include DON-glucuronides and DON-sulfates (52–54).

In light of the occurrence of the compounds and their mechanisms of action, it can be postulated that exposure to Uro A, AOH, and DON could affect intestinal functionality at several levels. Thus, this study aimed to decipher combinatory interactions of the foodborne substances, such as Uro A, AOH, and DON, taking multiple co-exposure scenarios into account. As food ingestion leads to exposure of the colonic epithelium, differentiated Caco-2 cells were chosen as a model for the evaluation of the repercussions of the compounds on intestinal metabolic capacity, and epithelial structural integrity. Finally, possible interactions of Uro A, AOH, and DON in the presence of a pro-inflammatory stimulus were also taken into account.

## Materials and Methods

### Chemicals and Reagents for Experiments

Cell culture media, supplements, and Dulbecco’s phosphate-buffered saline (DPBS) were bought at Gibco Thermo Fisher Scientific (Waltham, MA, United States). Plasticware for cell culturing and experiments was purchased at Sarstedt AG & CO (Nuembrecht, Germany), and ibidi (Graefeling, Germany). AOH [96%, contains 0.6% alternariol monomethyl ether (AME)], Uro A, CH223191 (CH-22), Benzo[*a*]pyrene (B[a]P), resorufin ethyl ether (7-ER), resorufin, dicoumarol, neutral red (NR) dye, bovine serum albumin (BSA) Fraction V, and Lucifer Yellow (LY) CH di-lithium salt fluorescent stain were obtained from Sigma Aldrich Chemie GmbH & Co (Steinheim, Germany), and DON from Romer Labs (Tulln, Austria). Dimethyl sulfoxide and Triton X-100 (TX) were bought at Carl Roth (Karlsruhe, Germany). Pierce™ Bicinchoninic Acid (BCA) Protein Assay Kit was obtained from Thermo Fisher (Waltham, MA, United States). Honeywell Ried-de Haën solutions: Liquid chromatography-mass spectrometry (LC-MS) grade water (H_2_O), methanol (MeOH), acetonitrile (ACN), and acetic acid were purchased at Fisher Scientific (Waltham, MA, United States). Antibodies for immunofluorescence experiments were purchased at Abcam and Santa Cruz: primary antibodies: α-tubulin (mouse; sc-5286), zona occludens 1 (ZO-1) (goat; ab190085), and CYP1A1 (rabbit; ab235185), and secondary antibodies: donkey anti-mouse, donkey anti-rabbit, and donkey anti-goat (Thermo Fisher Scientific).

### Cell Culture

Colon adenocarcinoma cell line Caco-2 (clone C2BBe1) was purchased from ATCC (Manassas, VA, United States) and cultured within Dulbecco’s Modified Eagle Medium (DMEM; Gibco), supplemented with 10% of fetal calf serum, 100 U/mL of penicillin and streptomycin, respectively, 1 mM of sodium-pyruvate, and 0.01 mg/mL of human insulin-transferrin-selenium. For experiments, they were used only at passage numbers below 30. The cells were seeded at a density of 85,000 cells/cm^2^ and allowed to grow until differentiated for 7 days as it was described previously (55–57). During this period, the medium was renewed every other day. The cancerous colonic cell line Caco-2 is known to form a polarized monolayer resembling the human intestinal structure (58), and was postulated to express the AhR, CYP1A1, and CYP1A2, all readily activated/inducible by AhR ligands (59, 60). Incubations for all experiments (exception: q-RT PCR) were carried out for 48 h. For experiments with IL-1β as a pro-inflammatory cytokine mediating intestinal inflammation, incubation solutions were spiked with IL-1β to an end-concentration of 25 ng/mL 2 h into the respective compound incubation. Substance stock solutions were prepared in either DMSO or dH_2_O, and solvent controls (SCs) were prepared accordingly. Applied substance concentrations were chosen based on previous reports in similar *in vitro* models, such as Caco-2 (7-day differentiated monolayers), or other colonic epithelial cell lines. Doses exerting no substantial cytotoxicity *per se*, yet responsiveness to inflammatory stimuli, were chosen for the study (AOH and Uro A 25 μM, DON 2.5 μM; 25, 56, 57).

### 7-Ethoxy-Resorufin-*O*-Deethylase Assay

7-ethoxy-resorufin-*O*-deethylase (EROD) assays for the determination of the capacity of the compounds to induce CYP1A1/1A2/1B1 enzyme activity in our cell system were conducted according to the method by Donato et al. (61). Cells were differentiated in 48-well tissue culture plates and incubated for 48 h. Subsequently, supernatants were aspirated and 7-ER EROD medium (10 μM) (DMEM no phenol red + 7-ER + dicoumarol) was added to the cell monolayer. Cells were incubated with EROD medium for 30 min. Immediately thereafter, 75 μL of EROD medium was transferred to 96-well plates, the wells already containing 200 μL of 99% EtOH, in triplicates. Fluorescence signal was measured at 535 nm_*ex*_/595 nm_*em*_ using a BioTek Cytation™ 5 Multi Mode Reader (Agilent Technologies, Santa Clara, CA, United States). Assessment of resorufin concentrations was conducted with the help of a resorufin standard curve, measured simultaneously. Afterward, cell monolayers were lysed *via* at least three freeze-thaw cycles (-80°C). For determination of protein content, a BCA assay was conducted according to the manufacturers’ instructions. Further, enzymatic activity was calculated using converted resorufin concentration, normalized to protein content. Two concentrations of B[a]P (1 and 5 μM) served as positive control for enzyme activity induction, whereas CH-22 (1 and 5 μM) served as an antagonistic control to inhibit enzyme activity (62, 63).

### Cell Viability Testing

For cell viability testing, Caco-2 cells were seeded as described in section “Cell Culture” and incubated for 48 h. To assess lysosomal activity, a NR assay according to Repetto et al. was chosen (64). For this purpose, a 4 mg/mL of NR stock solution in DPBS was prepared and diluted in a growth medium (4 μg/mL) and equilibrated at 37°C, 5% CO_2_, and 96% humidity for 24 h. Subsequent to the incubation time, the incubation medium was aspirated, cell monolayers were washed using warm DPBS, and 100 μL of NR medium was added and incubated for 3 h in the incubator. Afterward, cells were washed again in DPBS and 130 μL of destaining solution (99% EtOH: dH_2_O: glacial acetic acid 49.5:49.5:1) was added. Plates were shaken orbitally at 500 rpm for 10 min, and absorbance of supernatants was measured at 450 nm using a BioTek Cytation™ 5 Multi Mode Reader.

### Assessment of Transepithelial Electrical Resistance and Epithelial Permeability *via* Lucifer Yellow Assay

For transepithelial electrical resistance (TEER) measurements, cells were differentiated on Corning^®^ Transwell (TW) inserts (0.4 μm pore size) for 7 days. TEER values were measured after 24 and 48 h of incubation. Reported TEER values (Ω × cm^2^) were calculated according to the method of Srinivasan et al. (65). Subsequently, apical and basolateral medium was aspirated; however, cell monolayers were exposed to LY medium (Hanks Balanced Salt solution (66) buffer containing 0.1 mg/mL of LY) for 1 h. Afterward, the LY medium was aspirated and used for fluorescence measurements at a BioTek Cytation™ 5 Multi Mode Reader at 485 nm_*ex*_/535 nm_*em*_. The LY fluorescence was transformed into percentage of LY medium trespassing the cell monolayer and membrane resulting in permeability (%).

### Immunofluorescence Staining and Imaging

To determine the impact of the compounds on the protein expression of the tight junction protein ZO-1 and the Cytochrome P450 isoform CYP1A1, cells were seeded onto 8 well μ-dishes (ibidi). Subsequent to incubation (48 h), cell layers were washed twice using PBS-A and fixed with a 3.5% of formaldehyde solution (FA-Fix) in PBS-A for 15 min. FA-Fix solution was exchanged for PBS-A again, and slides were stored at 4°C until the staining procedure. Fixed cells were permeabilized (0.2% of TX in PBS-A), washed (PBS-A), and blocked (1% of BSA Fraction V in PBS-A). Primary antibody solutions were prepared as 1:250 dilutions in 0.25% of BSA in PBS-A and incubated for 2 h. After several washing steps (washing buffer: 0.02% of TX in PBS-A and PBS-A), secondary antibody solutions (1:1,000 dilutions in 0.25% BSA in PBS-A) were incubated for 1.5 h. Following further washing steps, antibodies were fixed (FA-Fix) for 10 min, cell monolayers were washed, and cells were quenched (0.75% m/v of glycine in PBS-A) for 10 min. Stained cells were embedded in Mounting Medium with DAPI, Aqueous Fluoroshield (ab104139, Abcam) and stored at 4°C until imaging. Images were obtained using a LSM Zeiss^®^ 710 microscope coupled to an ELYRA PS.1 system, equipped with an AndoriXon 897 (EMCCD) camera and a Plan Apochromat 63X objective. Immunofluorescence experiments were performed in at least three biological replicates and at least five technical replicates consisting of randomly chosen areas within each well of the microscopy slides. Image analysis was conducted using Zeiss Imaging and Analysis software ZEN (black edition), randomly selecting at least 5 regions of interest (= 5 technical replicates per biological repetition). Fluorescence intensities for each channel were then normalized to the solvent control condition.

### Liquid Chromatography Tandem Mass Spectrometry Analysis of Urolithin A, Alternariol, and Deoxynivalenol Parent Compound Recoveries and Their Metabolites Produced and Secreted by the Cells

To evaluate passage and metabolism of the apically applied compounds, cell monolayers in the TW system were exposed to the substances according to the procedure described in section “Assessment of Transepithelial Electrical Resistance and Epithelial Permeability *via* Lucifer Yellow Assay.” However, after incubation (48 h), the apical and basolateral medium was aspirated, immediately quenched (-20°C, supernatant: ACN 50:50), and stored at -80°C until further processing for LC-MS analytical purposes. Meanwhile, cell monolayers were lysed and quenched (ACN : MeOH : H_2_O (40 : 40 : 20), 4 freeze-thaw cycles using liquid N_2_), and stored at -80°C until further processing. For LC-MS analysis, the samples were thawed, centrifuged (18,000 × *g* at 4°C), and transferred into glass vials. The LC-MS analysis was conducted using a 1290 Infinity II LC System (Agilent Technologies, Waldbronn, Germany) coupled with a QTrap 6500+ (AB Sciex, Redwood City, CA, United States). A previously published method (67) was adapted to the needs of the three different substances and their metabolites. Both, the operation of the LC-MS/MS system and further data analysis were performed using Analyst 1.7.0 software.

To obtain chromatographic separation of Uro A, AOH, DON, and their respective metabolites, a previously published method was used as a starting point (67). The MS/MS parameters of the newly implemented compounds were optimized by syringe injection. Method optimization was applied to serve the necessities of the diverse structures. Samples were injected into a Kinetex^®^ Biphenyl 100 Å column (150 mm × 3.0 mm, 2.6 μm, Phenomenex, Aschaffenburg, Germany) equipped with a guard column of the same type at 40°C. The mobile phases used contained the following mixtures: eluent A: 10% of MeOH and 0.05% of AA in H_2_O (LC-MS grade) and eluent B: 0.05% of AA in MeOH (LC-MS grade). The flow rate was set to 0.4 mL/min. An initial phase of 1 min pure eluent A was followed by a linear increase of eluent B reaching 16% after 10 min, 40% after 11 min, and further to 100% B after 14 min. After column flushing for 3 min with 100%, the column was equilibrated with the starting conditions resulting in a total run time of 20 min. The default injection volume was set to 2 μL. The QTrap 6500+ was operated in the negative ionization mode using a Turbo Spray IonDrive ion source with the following parameters: curtain gas (CUR, nitrogen), 35 psi (241 kPa), collision gas (CAD, nitrogen), high ion spray (IS) voltage of -4,500 V, temperature of 451°C, and sheath gas (GS1) and drying gas (GS2) of 60 psi (414 kPa, zero grade air). Multiple reaction monitoring (MRM) parameters operated in the scheduled MRM mode applied for all compounds analyzed can be found in Table 1. Enhanced product ion (EPI) scans of compounds and metabolites identified can be found in the Supplementary Figures 6–17.

**TABLE 1:** Multiple reaction monitoring (MRM) parameters for liquid chromatography tandem mass spectrometry (LC-MS/MS) data evaluation.

Analyte	Retention time (min)	Precursor ion (m/z)	Declustering potential DP (V)	Product ions*^a^* (m/z)	Collision energy CE*^a^* (V)	Cell exit potential CXP*^a^* (V)	Entrance potential EP (V)	Dwell time (ms)	Ion ratio qualifier: quantifier
Uro A	14.3	226.9	–115/–115	197.9/183.0	–44/–32	–11/–13	–10	15	0.42
Uro A-S	14.4	306.9	–20/–20	227.0/197.9	–25/–70	–11/–11	–10	15	
Uro A-GlcA	14.2	403.1	–60/–60	227.0/197.9	–40/–90	–11/–11	–10	15	
AOH	14.7	257.0	–100/–100	215.0/213.0	–36/–34	–11/–11	–10	15	0.79
AOH-3-S	15.4	337.0	–40/–40	257.1/213.0	–40/–50	–10/–10	–10	15	0.23
4-OH-AOH	14.4	273.0	–60/–60	214.2/258.1	–40/–30	–11/–11	–10	19.8	0.16
AOH-GlcA	14.7	433.1	–40/–40	257.0/215.0	–50/–70	–11/–11	–10	15	
AME	15.8	271.1	–95/–95	227.0/256.0	–32/–50	–13/–11	–10	22.5	0.19
AME-3-S	15.1	351.0	–60/–60	271.1/256.0	–50/–50	–10/–10	–10	15	
4-OH-AME	15.6	287.1	–40/–40	272.1/228.1	–30/–30	–11/–11	–10	16.6	
DON	10.0	355.1	–50/–125	59.2/265.2	–24/–24	–13/–13	–10	22.9	0.03
DON-3-S	9.8	345.0	–125/–125	345.0/97.0	–36/–36	–21/–21	–10	33.1	
DON-15-S	8.5	375.0	–110/–110	97.0/345.0	–50/–50	–9/–9	–10	33.1	
DON-3-GlcA	9.5	471.1	–60/–60	113.0/175.1	–40/–40	–12/–12	–10	22.6	
DON-15-GlcA	9.4	471.0	–60/–60	193.1/265.0	–30/–30	–12/–12	–10	22.2	

*^a^Quantifier/qualifier.*

### Quantitative Real-Time PCR

To investigate the relative gene expression of the CYP1A1 isoform, quantitative real-time PCR (q-RT PCR) was performed after 6 h of incubation. For a minimum of four individually conducted experiments, the cells were seeded as described into 24-well plates, incubated for 6 h and subsequently processed for messenger RNA (mRNA) purification utilizing a Maxwell^®^ 16 LEV simplyRNA Cells Kit from Promega. According to the manufacturer’s protocol, cell monolayers were lysed and harvested for RNA extraction. Afterward, RNA purity and concentration were measured using a NanoDrop™ 2000/2000c spectrophotometer (Thermo Fisher Scientific) and frozen to -80°C until continuation. RNA (1 μg) was transcribed into complementary DNA (cDNA) using a QuantiTect^®^ Reverse Transcription Kit (Qiagen, Hilden, Germany) following the manual’s instructions, and stored at -20°C until further processing. QuantiTect^®^ SYBR^®^ Green Master Mix and gene specific QuantiTect^®^ Primer Assays (Qiagen, Hilden, Germany) were used for DNA amplification using a StepOnePlus™ System (Applied Biosystems). Primer assays for the genes of interest were the following: hypoxanthine phosphoribosyltransferase 1 (hPRT1): Hs_HPRT1_1_SG; QT00059066; delta-aminolevulinate synthase (ALAS): 1Hs_ALAS1_1_SG; QT00073122; and Cytochrome P450 1A1 (CYP1A1): Hs_CYP1A1_1_SG; QT00012341; ALAS and hPRT1 were utilized as endogenous control genes. Calculation of relative gene expression levels was conducted applying the 2^−ΔΔ^CT method, described by Livak and Schmittgen (68).

### Data Evaluation and Statistical Analysis

All the above-mentioned experiments were conducted in a minimum of 3 independent workflows (information on technical replicates can be found in the respective “Materials and Methods” section). Data were summarized and collected in Excel 2016. Statistical analysis was conducted in Origin Pro 2020. Statistical tests applied for each experiment can be found in the respective “Results” section and Figure captions.

## Results

### Impact on CYP1A1/1A2/1B1 Metabolic Enzymes (EROD Assay)

**FIGURE 2:**
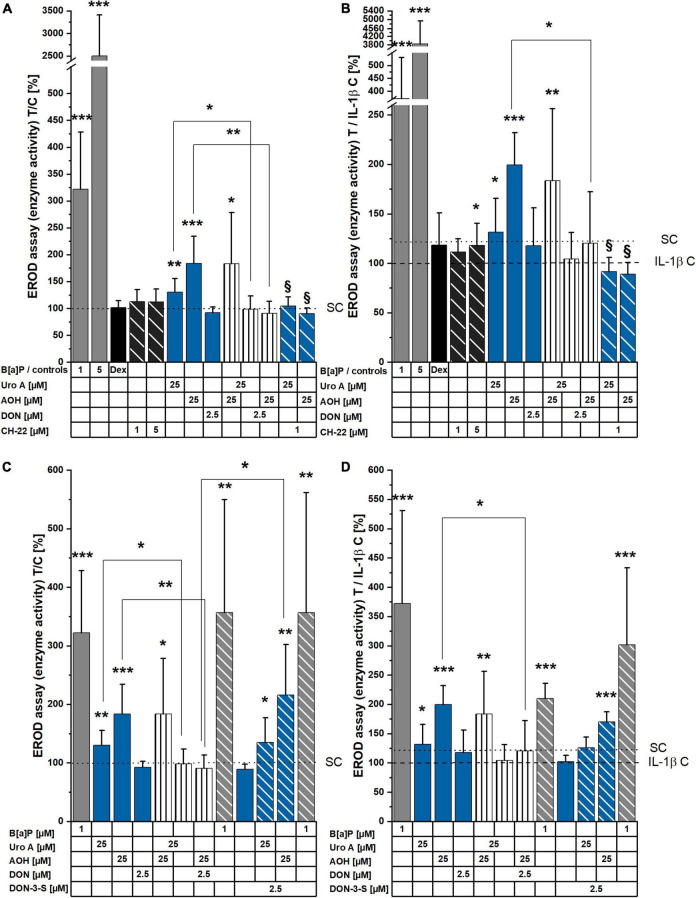
7-ethoxy-resorufin-*O*-deethylase (EROD) assay measurements showing induction of CYP1A1/1A2/1B1 enzyme activity. **(A,B)** Cell monolayers were incubated with compounds for 48 h, and **(C,D)** additionally stimulated with IL-1β (25 ng/mL), 2 h into substance incubations. Enzyme activity (pmol resorufin/30 min/protein) was normalized to the respective control condition and is presented as T/C in%. Significant differences compared to the controls were calculated applying two-sample Students’ *t*-test and are shown as *p < 0.05, **p < 0.01, and ***p < 0.001. ^§^ marks significant differences of combi incubations with CH-22 compared to the respective single compound (*p* < 0.05).

To investigate the effects of the dietary compounds, Uro A, AOH, and DON on intestinal cells, experiments were performed to assess the activation of the AhR signaling pathway. Activated AhR signaling is recognized to regulate cell metabolism and can serve as the central hub for mechanisms necessary for the maintenance of the epithelial barrier function (11, 12, 15, 17). Hence, EROD assays were conducted to elucidate the potential of the molecules to induce CYP1A1/1A2/1B1 enzyme activity, and therefore measure the capacity of the compounds to activate the AhR pathway at the very end of the signaling cascade. The known agonist of AhR, B[a]P, and antagonist of the complete signaling cascade CH-22 were used as positive and negative controls, respectively (62, 63, 69). Dexamethasone (Dex) was included as a reference anti-inflammatory drug (70). Responsiveness of Caco-2 cells to the positive control B[a]P was dose dependent (1, 5 μM), while CH-22 and Dex showed no effect (Figure 2A). Incubation (48 h) with Uro A and AOH (both 25 μM) as single compounds and in a binary mixture led to enzyme activity induction, revealing AOH to be more potent (Figure 2A). Their binary mixture showed rather additive interactions between the two compounds, as the level of the enzyme activation was comparable to AOH alone. DON, as single compound, did not activate the AhR pathway. When Uro A and AOH were co-incubated with 2.5 μM of DON, the responses of the two DAPs alone were reduced, respectively. The same could be observed for the combined incubations with the inhibitor CH-22 (Figure 2A). To check, whether the effect of DON on the efficacy of the two DAPs could be traced back to its proteostatic effect, DON was exchanged for DON-3-sulfate (DON-3-S), which is unable to bind into the ribosomal pocket, and hence, cannot reproduce the block of the protein synthesis triggered by the parent compound (71, 72). Indeed, when the substances were co-incubated with 2.5 μM of DON-3-sulfate instead of DON, the CYP1 enzyme activation by Uro A and AOH was again observed.

To mimic an inflammatory scenario in the colon, IL-1β (25 ng/mL) was applied to the experimental setup 2 h into substance incubation and led to a reduced basal level of CYP enzymes activity compared to the solvent controls (Figure 2B). Concerning the controls, B[a]P and CH-22 maintained roughly the same efficacy profile compared to the non-inflammatory experiment. Similarly, single, and combinatory Uro A and AOH induced EROD enzyme activity, and this effect was reduced by the presence of DON. Within the inflamed system, DON also reduced the enzyme activation triggered by B[a]P. The EROD activities reduced by DON as combinatory partner could be observed again with DON-3-S as substitute for DON in combination with Uro A, AOH, and B[a]P (Figure 2D).

### Cytotoxicity Assessment (NR Assay)

**FIGURE 3:**
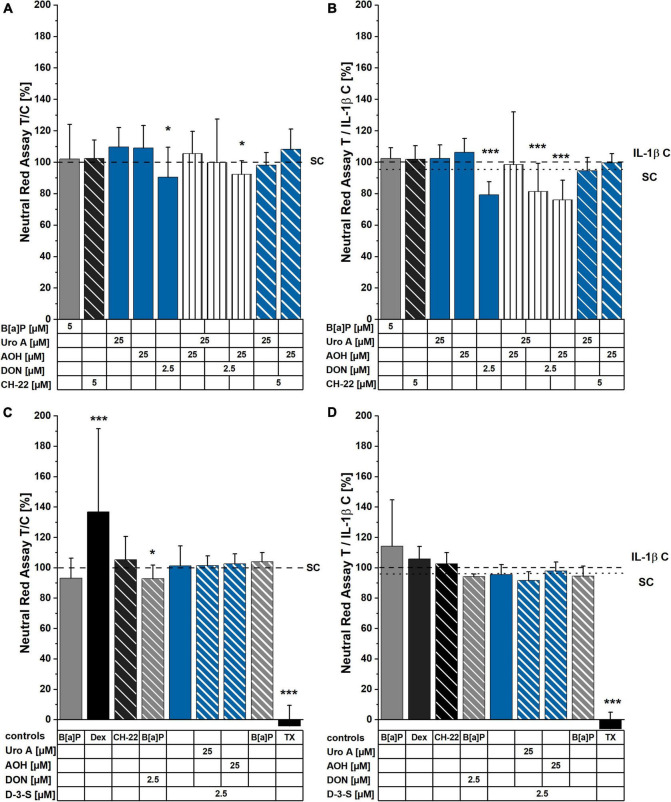
Neutral Red (NR) assay after 48 h of incubation. Cells were either exposed to compounds for 48 h **(A,C)**, or stimulated with IL-1β, 2 h into substance incubation **(B,D)**. Differences in incubation conditions shown in **(A,B)** compared to **(C,D)** concern B[a]P: 5 μM **(A,B)** and 1 μM **(C,D)**. Dexamethasone (Dex) was applied at 5 μM **(C,D)**. Lysosomal activity measurements are normalized to solvent control or IL-1β control, respectively. For significant differences compared to control, two-sample Students’ *t-*test was applied and is marked by asterisks: *p < 0.05, ***p < 0.001.

The NR assay was applied to measure cell viability/lysosomal activity within 48 h of incubation with the compounds of interest. With exception of a limited reduction of cell viability for the cells incubated with 2.5 μM of DON and 2.5 μM of DON + 25 μM of AOH, no significant changes in cell viability were observed. In the presence of pro-inflammatory stimulation (25 ng/mL IL-1β 2 h into incubation with the compounds), however, the toxicities of 2.5 μM of DON, as well as of the binary mixtures of AOH and Uro A with DON were significantly enhanced (∼80 ± 4% cell viability; Figures 3A,B). In contrast to DON, its human metabolite DON-3-S exerted no toxicity in any of the experimental conditions tested (Figures 3C,D).

### Cell Monolayer Integrity (TEER)

**FIGURE 4:**
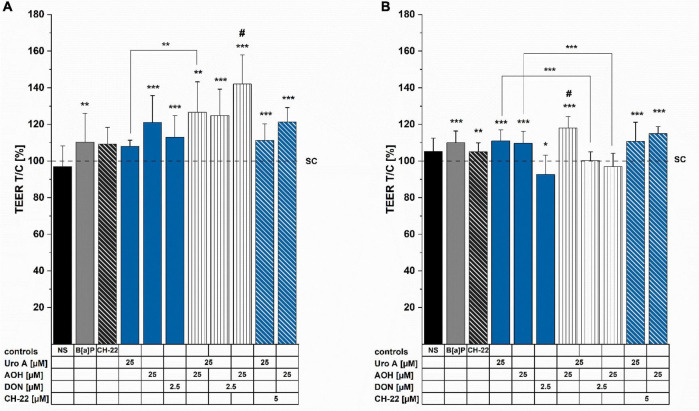
Transepithelial electrical resistance (TEER) measurements were conducted after **(A)** 24 h and **(B)** 48 h of incubation. TEER_*REPORTED*_ values were normalized to the solvent control condition and are presented as percentages. Significant differences were calculated applying two-samples Students’ *t-*test, at *p < 0.05, **p < 0.01, ***p < 0.001; ^#^*p* < 0.05 represents significant differences of binary mixtures compared to the respective single compounds. NS 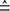 no solvent control.

Activation of the AhR was reported to support the maintenance of epithelial barrier architecture, which is crucial for intestinal homeostasis (12). Hence, TEER measurements were conducted to provide information on the potential effects of the foodborne compounds on the cell monolayer integrity in our *in vitro* model. Incubation for 24 h enhanced TEER for the AhR activating positive control of 5 μM of B[a]P, as well as the single compounds, AOH (25 μM) and DON (2.5 μM; Figure 4). All binary mixtures of the compounds led to significantly elevated TEER values. Intriguingly, the combination of AOH and DON showed significantly increased TEER values compared to both single toxins, respectively. Similar induction could also be observed for Uro A + AOH; however, it was only significantly different compared to Uro A alone (Figure 4A). Besides, although the AhR antagonist, CH-22 (5 μM) led to no substantial alterations in TEER values after 24 h, combinatory mixtures of Uro A and AOH with the inhibitor still elevated TEER measurements. Substance exposure for 48 h significantly enhanced TEER values for both controls, 5 μM of B[a]P and 5 μM of CH-22. Furthermore, single incubations with Uro A or AOH increased TEER values, while DON triggered significant reduction in TEER (Figure 4B). The binary mixture, Uro A + AOH significantly enhanced TEER values even further compared to the single compounds. Combinations of DON with Uro A and AOH diminished the effects of both single DAPs, respectively (Figure 4B).

### Cell Monolayer Permeability (LY Assay)

**FIGURE 5:**
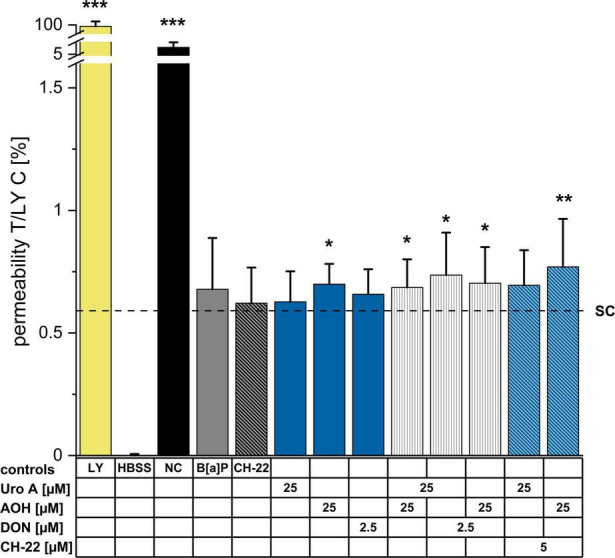
Lucifer Yellow (LY) dye-assisted permeability measurements. Passage of LY through the differentiated Caco-2 monolayer was measured after 48 h of incubation (at least 3 biological replicates) by adding LY dye to the apical side of the monolayer for 1 h. LY (0.1 mg/mL in HBSS buffer), HBSS (blank), no cells (NC, passage through membrane only); Fluorescence of pure LY dye solution was set to 100%. Permeabilities as reported by fluorescent signals are presented as percentages. Statistical analysis was calculated applying two-sample Students’ *t*-test; significant differences are shown as *p < 0.05, **p < 0.01, or ***p < 0.001 against the solvent control.

To complement the information obtained with the TEER measurements, LY assay was performed to determine the impact of the compounds on epithelial permeability after 48 h of incubation (Figure 5). Compared to the solvent control, the passage of LY dye was moderately (not significantly) enhanced by B[a]P (5μM) and CH-22 (5μM). Regarding the exposure to single food-derived substances, AOH moderately, yet significantly increased the permeability of the cell monolayer, while Uro A (25 μM) and DON (2.5 μM) showed no effect. The binary mixture Uro A + AOH enhanced the passage of LY dye to a similar extent as AOH alone. An increased permeability through the Caco-2 monolayer could be observed after the incubation with combinations of both Uro A and AOH together with DON. The capacity of AOH to allow permeation of LY dye was further enhanced by co-incubation with CH-22. An elevating tendency (yet not significant) could be seen for Uro A + CH-22 as well.

### Tight Junction Scaffold Protein ZO-1 Immunolocalization

**FIGURE 6:**
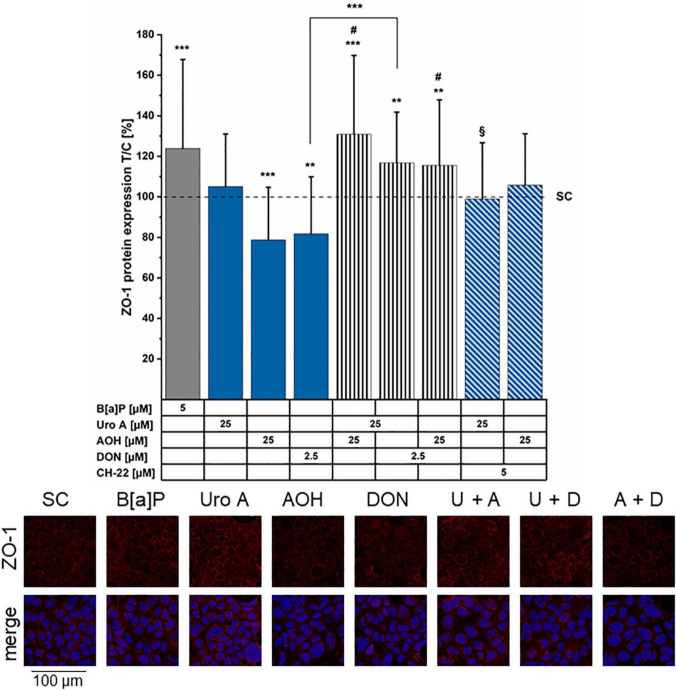
Immunofluorescence staining of ZO-1 protein. ZO-1 was stained after 48 h of incubation period (minimum 4 biological replicates). Fluorescence intensities were normalized to the solvent control (SC) and are presented as test over control (T/C) in percent. Significant differences compared to the solvent control (SC) were calculated by applying two-samples Students’ *t*-test and are shown as *p < 0.05, **p < 0.01, and ***p < 0.001. Significant differences of binary combinations compared to both single compounds are marked with ^#^*p* < 0.01. ^§^*p* < 0.01 marks differences of combi incubations with CH-22 compared to the expression level of the respective second compounds. In the example images, the abbreviations U for Uro A, A for AOH, and D for DON are used for the combinations. ZO-1 (red) shows ZO-1 immunofluorescence signal only, merge shows the combination of ZO-1 + nucleus (blue).

To gain an overview of the molecular mechanisms potentially supporting the variations of cell permeability, immunofluorescence experiments were performed to investigate the localization of junctional proteins regulating intestinal cell–cell contacts. The tight junction scaffold protein, ZO-1 was chosen as it serves as an essential parameter for the epithelial barrier integrity and differentiation of the cell monolayer (73). In accordance with the TEER measurements (Figures 4A,B), B[a]P (5 μM) increased ZO-1 protein immunolocalization (Figure 6). AOH (25 μM) and DON (2.5 μM) reduced ZO-1 expression levels in comparison to the solvent control. Uro A, as single substance showed no impact on ZO-1 fluorescence intensity; however, the binary mixture, Uro A + AOH significantly enhanced ZO-1 expression. Interestingly, the combinations of AOH + DON and of Uro A + DON, increased ZO-1 protein expression; nonetheless, to a reduced extent in comparison to the combination AOH + Uro A. Combinatory incubation with CH-22 put ZO-1 fluorescence with AOH back to a level comparable to the solvent control. No impact was found for CH-22 on Uro A in this context. Alterations in ZO-1 appearance can be seen from the example images (Figure 6, image panel). Images not included in the primary manuscript can be found in the Supplementary Figure 1.

### Liquid Chromatography Tandem Mass Spectrometry Analysis

**FIGURE 7:**
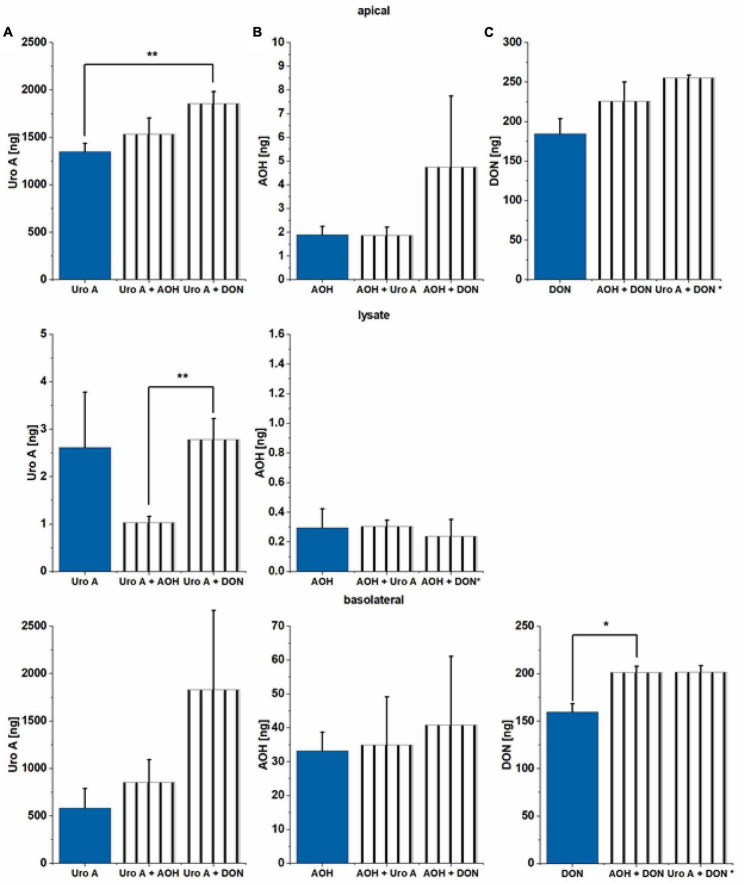
Liquid chromatography tandem mass spectrometry (LC-MS/MS) analysis of apical and basolateral media and lysate content. **(A)** Uro A, **(B)** AOH, and **(C)** DON were measured after 48 h of incubation in the Transwell (TW) system. Recoveries (%) are related to the parent compound (ng) applied to the apical side of the Caco-2 monolayer. Effectively applied amounts: Uro A: 2,850 ng, AOH: 3,230 ng, and DON: 370 ng. Significant differences between incubation conditions were calculated applying Students’ *t*-test, and are marked by *p < 0.05, and **p < 0.01; * in the y-axis indicates the respective bar to depict *n* = 2 biological replicates.

Alterations of epithelial barrier properties are known to impact the transport of exogenous chemicals through the intestinal epithelium (74). Thus, apical and basolateral media, as well as cell lysate components were examined *via* LC/MS-MS to explore substance absorption, passage, or metabolism rates after application to the apical side of the Caco-2 differentiated monolayer in the TW system. Incubation (48 h) with Uro A (25 μM) correspond to an application of effectively 2,850 ng of Uro A to the cell monolayer. In total, Uro A was recovered in all three compartments tested to a minimum of 1,940 ng (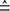
68 ± 9% of substance applied). Combinatory incubations of Uro A with AOH and DON led to even higher recovery rates (Uro A + AOH: 2,390 ng Uro A 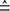 84 ± 10%; Uro A + DON: 3,690 ng Uro A 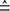 129 ± 25%; Figure 7A). For all three conditions, the largest amount recovered Uro A was found in the apical compartment, except for the binary mixture of Uro A + DON, which led to nearly the same recovery apically and basolaterally (Figure 7A). Moreover, we could recover Uro A in the lysate for all three conditions as well, Uro A + AOH resulting a significantly lower recovery rate compared to the other two incubation scenarios. About 25 μM of AOH resulted in effectively incubating cells with 3,230 ng, of which only a small amount was found in the three media investigated (Figure 7B). The single incubation with AOH enabled in 35.4 ng of AOH recovery (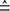
1.1 ± 0.2%), while the binary mixtures led to the following recoveries: AOH + Uro A: 37.2 ng AOH (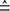
1.2 ± 0.5%), AOH + DON: 45.9 ng AOH (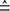
1.4 ± 0.7%). AOH was found in all three compartments, while most of the recovered AOH was observed in the basolateral media for the three different exposure scenarios. Of note, the distribution of the recovered shares was found to be significantly different for the binary mixture of AOH + DON. This exposure scenario led to a larger share within the apical media compared to the other incubation conditions (Supplementary Figure 2). Apical exposure to 2.5 μM of DON resulted in effective incubation with 370 ng of DON. LC-MS/MS measurements enabled high recovery of DON in two of the tested compartments for the three conditions (Figure 7C). DON was found in the apical and the basolateral media investigated, resulting in nearly the same share for both compartments, yet higher recovery rates within the apical media (Figure 7C). Incubating DON as single compound led to a recovery of 345 ng (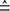
93 ± 3%), while combinations led to higher recoveries: AOH + DON: 427 ng DON (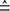
115 ± 8%), Uro A + DON: 457 ng of DON (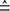
123 ± 1%). Low recoveries in lysates made quantitative analysis impossible for this compartment. Overall, quantitatively determined metabolites include AME and AOH-3-sulfate (AOH-3-S; Supplementary Figure 3A); however, AME was measured to be present in the applied AOH (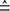
20 ng AME of 3,230 ng of AOH applied; 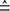
0.6%; data not shown). Due to low abundance, recoveries between the limit of detection and the limit of quantification, or missing reference standards (hence, tentatively annotated), metabolites found, yet not quantitatively analyzed included 4-OH-AOH, AOH-GlcA, AME-3-S, Uro A-S, and Uro A-GlcA. AME could be quantitatively determined in all three compartments (Supplementary Figure 3B). The biggest share of AME was found in the basolateral media for all three incubation conditions (∼90 ± 2% of total recovery), the total recovery ranging from 1.0–1.3 ng within the three exposure scenarios (Supplementary Figure 3B). AOH-3-S was found in all three compartments, the biggest share recovered in the apical media for all tested conditions. Total AOH-3-S recovery for single incubation of AOH reached 348 ng, for AOH + Uro A: 361 ng, and for AOH + DON: 436 ng (Supplementary Figure 3). Metabolites of Uro A and AOH, that could not be quantitated, yet their relative recoveries (normalized to Uro A single incubation condition) are shown in Supplementary Figures 4, 5. EPI scans can be found in Supplementary Figures 6–17.

### Relative Gene Transcription Levels, and Immunofluorescence Imaging of CYP1A1 Protein

**FIGURE 8:**
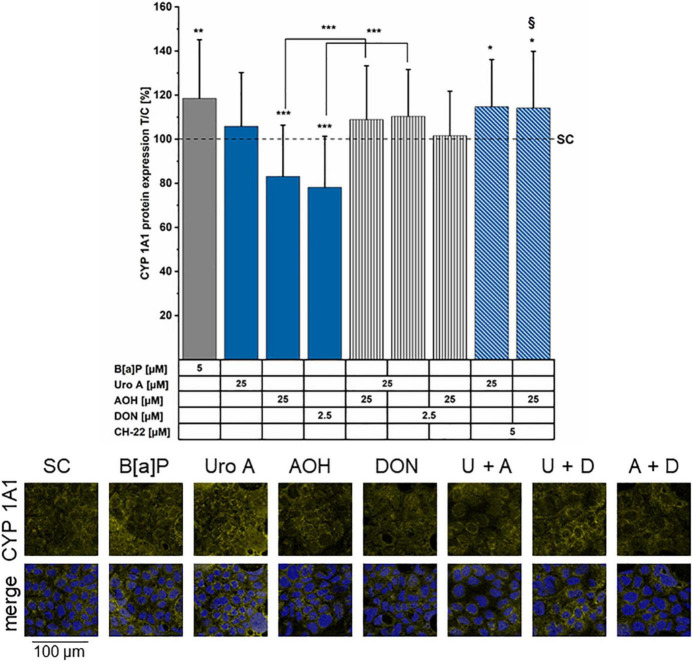
Immunofluorescence staining of CYP1A1 protein. CYP1A1 was stained after 48 h of incubation period (minimum four biological replicates). Fluorescence intensities were normalized to the solvent control (SC) and are presented as test over control (T/C) in percent. Significant differences compared to the SC were calculated by applying two-samples Students’ *t*-test and are shown as *p < *0.05*, **p < *0.01*, and ***p < *0.001*. ^§^*p* < *0.01* marks differences of combinatory incubations with CH-22 compared to the respective second compound. Panel of immunofluorescence images showing CYP1A1 protein localization (yellow) in the following conditions: SC, B[a]P 5 μM, Uro A 25 μM, AOH 25 μM, and DON 2.5 μM. Merge shows CYP1A1 (yellow) and nuclei (blue) together. In the example images the abbreviations U for Uro A, A for AOH, and D for DON are used for the combinations.

As key molecular pathways downstream of the AhR activation involve metabolic competence (75), we proceeded to evaluate the potential effects of Uro A, AOH, and DON on CYP1A1. Transcription was investigated *via* q-RT PCR after 6 h of exposure to B[a]P, Uro A, and AOH. The positive control for AhR pathway activation, B[a]P 5 μM triggered a 26 ± 15-fold elevated transcription level of CYP1A1. On the contrary, AOH and Uro A (25 μM) as single compounds and the binary mixture (1:1) suppressed the transcription of CYP1A1 relative to the solvent control at this time point: AOH (0.07 ± 0.02 fold), Uro A (0.17 ± 0.03 fold), Uro A + AOH (0.10 ± 0.03 fold; Supplementary Figure 18). As complementary analysis to the q-RT PCR, immunofluorescence experiments were performed for the localization of CYP1A1 after 48 h of incubation. Fluorescence intensity measurements (Figure 8, image panel) showed enhanced CYP1A1 protein localization after incubation with the AhR positive control B[a]P (5 μM). In line with the inhibitory effect on protein biosynthesis, DON reduced the expression of CYP1A1. Incubation with AOH (25 μM) significantly reduced CYP1A1 expression levels, whereas Uro A (25 μM) did not alter CYP1A1 immunolocalization. Concerning binary mixtures, all combinations re-established the CYP1A1 expression level no different than the solvent control. However, also AOH and DON in combination showed protein expression to be restored, or even slightly (yet not significantly) enhanced as compared to the solvent control. Besides, in the presence of the inhibitor, CH-22, the incubation with AOH and Uro A resulted in CYP localization levels compared to those of the positive control B[a]P.

## Discussion

The present study focusses on the impact of foodborne xenobiotics on colonic cell metabolic competence, epithelial barrier functions, and absorption patterns. In this context, the role of the AhR pathway in maintaining intestinal barrier integrity, and subsequently Phase I- and Phase II-metabolism were investigated. For higher relevance of the potential exposure scenarios, the activities of the ellagic acid metabolite, Uro A and the two mycotoxins, AOH and DON were examined as single compounds along with binary combinations. For the first time, to the best of our knowledge, this work provides insights on DON acting differently on the colonic epithelial barrier integrity and its metabolic capacity compared to Uro A and AOH; furthermore, DON dominates the absorptive and metabolizing behavior of the cells to binary exposures in this context. In contrast, the two DAPs Uro A and AOH dominate the sustainment of the epithelial structural integrity in Caco-2 cell monolayers, in particular in combinatory exposure scenarios.

Exogenous and endogenous small molecules characterized by a planar scaffold are known to activate the AhR pathway. This reflects on cellular metabolism (CYP enzymes), immune response, and epithelial homeostasis (structural proteins turnover) in the gut (76, 77). Colonic cancer cells were found to express higher levels of CYP1A1/1B1/2E1 and GST isoforms in comparison to a healthy colonic epithelium, suggesting this signature to be a useful marker for the prognosis of cancer progression (78). Hence, we investigated the potential of the microbial metabolite, Uro A, and the mycotoxins, AOH and DON to induce CYP1A1/1A2/1B1 enzyme activation *via* EROD assays (Figures 2A–D). A recent study reported AhR activating *Alternaria* toxins within complex mixtures to interact synergistically upon CYP 1 family activation (79). Nonetheless, the effect of the binary mixture of Uro A and AOH did not exceed the efficacy of single toxins (Figures 2A,B) in our Caco-2 cell model. In line with our results, Uro A was recently described as AhR ligand; however, it could either support or antagonize 2,3,7,8-tetrachlorodibenzo-*p*-dioxin (TCCD) induced activation of the AhR, depending on the *in vitro* model used (26). In turn, exposures to mixtures with DON diminished the potencies of single DAPs in this respect (Figures 2A,B). However, when exposed to the DON human colonic metabolite, DON-3-S (67) instead of DON, Uro A and AOH could exert their EROD activating capacities, respectively (Figures 2C,D). Hence, the suppressive effect of DON could be directly linked to its capability to inhibit protein synthesis. This interpretation finds confirmation in the cell viability tests (Figures 3A–D). DON reduced NR uptake alone, as well as in combination with AOH. However, in cells additionally stimulated with IL-1β, DON led to a significant reduction in the lysosomal activity as single compound, and in binary mixtures with AOH and Uro A (Figures 3A,B), possibly potentiating cellular stress due to the inflammatory stimulus. Intriguingly, its human metabolite, DON-3-S displayed no such effects, neither alone, nor in binary mixtures (Figures 3C,D). The NR uptake assay captures the ability of the cells to maintain pH gradients, membrane homeostasis, and functionality of lysosomes (80). As such, this assay could highlight DON to exert slight, yet significant, cytotoxicity, an effect even more pronounced in further stressed cells, due to inflammatory triggers (Figure 3B). This is in line with previous reports on DON exerting cytotoxic effects in a dose-dependent manner in differentiated Caco-2 cells (56), as well as epidermal carcinoma cells A431 (81), and targeting lysosomal homeostasis (82). Nonetheless, the cytotoxic effect was shown to comply even more distinctly in proliferating cells, compared to differentiated ones like the Caco-2 cells (83). Considering a more physio-pathological context, mediators of inflammatory responses, such as the pro-inflammatory cytokine IL-1β, are known to trigger signaling cascades like the NF-κB pathway, which was reported to cross-talk with AhR signaling (84). Indeed, also in our experimental setup, Caco-2 monolayers stimulated by 25 ng/mL of IL-1β showed reduced EROD enzyme activity levels compared to reference activities in solvent controls (Figures 2B,D). Moreover, Uro A and AOH, previously reported for their immunomodulatory potential, readily induced CYP1A1/1A2/1B1 enzyme activity to similar extents compared to the respective control (Figures 2B,D). DON affected EROD enzyme activities slightly different after inflammatory stimulus compared to without inflammatory triggers. This should be interpreted under the premise, that it was described to rather exacerbate intestinal inflammatory responses involving the NF-κB pathway in Caco-2 cells and other intestinal *in vitro* models at similar concentration levels as applied here (51, 56, 85).

In malignant intestinal tissues, the AhR expression is reportedly predominant within the epithelial cells, as well as immune cells located in the lamina propria (86, 87). Hence, its importance in several GIT- related pathological conditions by maintaining tissue homeostasis is regularly demonstrated (88–90). TEER measurements presented in this study (Figures 4A,B) revealed the compounds of interest to exhibit time-dependent effects toward Caco-2 monolayer integrity. Exposure to the AhR positive control B[a]P as well as AOH and Uro A resulted in elevated TEER values. The trend towards increasing TEER for the two DAPs was sustained in their binary exposure scenario (Uro A + AOH) (Figures 4A,B). In line with our findings, a recent study described a dose-dependent increase in Caco-2 TEER values for Uro A. Besides, Uro A was described to counteract a TNF-α induced loss in epithelial barrier integrity in a HT-29/B6 co-culture model, which was accompanied by maintenance of basal levels or reduction of protein expression levels of claudin 1 and claudin 2, respectively, which were both upregulated upon TNF-α stimulation (91). However, DON significantly reduced TEER values, and could reduce the effects exerted by both DAPs, when co-incubated (Figure 4). Slightly enhanced LY permeability was observed after exposure to AOH and Uro A + AOH (Figure 5). DON combined with Uro A and AOH increased the relative amount of permeating LY. In line, DON was previously reported to disrupt epithelial barrier integrity *in vitro* (Caco-2 cells, clone: HTB-37) and *in vivo* (male B6C3F1 mice) at similar concentration levels already after 8–12 h. DON exposure reduced tight junction protein levels and altered their localization. Concomitantly increasing mRNA transcription for tight junction and scaffold proteins, this effect was linked to the protein biosynthesis inhibiting capacity of DON (92). Of note, recurring exposure to high levels of DON was associated with a detrimental loss of barrier integrity, which was accompanied by substantial passage of bacteria to the basolateral compartment *in vitro* (49). In our experimental setup, we observed both AOH and DON to reduce ZO-1 immunofluorescence signals (Figure 6). Even if data on AOH are limited, a similar concentration of DON (2 μM) was suggested to induce epithelial barrier disruption by the degradation of tight junction proteins *via* p38 MAPK and JNK signaling (93), p38 MAPK being recognized as one pathway triggered by low doses of DON contributing to its pro-inflammatory effects (94). Combinatory exposure to Uro A and AOH led to enhanced ZO-1 immunolocalization (Figure 6). This finding is supported by a recent report of Uro A to induce tight junction protein expression in several colonic *in vitro* and *in vivo* models preserving the intestinal barrier function against inflammatory stress. At the molecular level, this was linked to its AhR and Nrf-2 pathway activating potential (25). Concerning their binary mixture effects, Uro A seems to dominate the effect toward ZO-1 protein expression. ZO-1 is a TJ protein considered as a parameter for Caco-2 (HTB-37) differentiation status (73, 95). Conversely, AOH possibly dictates the overall impact on TEER and LY, both serving as measures of barrier integrity *per se* (Figures 4, 5). Of note, changes in TEER during ongoing differentiation post-confluency were recently connected to alterations in cell circumferential junctional length (lining of junctional proteins along the cell–cell contacts) as well, while permeability was not further altered (96). However, other proteins of the apical junctional complex (97) could be involved in the effects seen toward the barrier integrity by AOH (Figures 4, 5) as well, as it was described for Uro A (25). Co-exposure of CH-22 with AOH re-established the ZO-1 immunofluorescence signal of the cells to the level of the solvent control (Figure 6). This is accompanied by a restoration of the EROD assay signal to values similar to those of controls (Figures 2A,C). This strongly infers for the involvement of the AhR activation in the effects of the DAPs at the intestinal level. Even if CH-22 showed a distinct efficacy toward the endpoints investigating the epithelial barrier structural integrity (Figures 4–6), this could be partly explained by a complementary mechanism. The molecule was previously reported to counteract ROS production and lipid peroxidation (LP) accompanied by AhR and CYP1A1 activation (98). Elevated ROS and LP, however, were both associated with epithelial barrier disruption proceeding as either reduced TEER or enhanced permeability of Caco-2 monolayers (99). From this perspective, it is worth mentioning that both Uro A and AOH were previously reported to hold the potential to modulate cellular redox status (27, 100–102).

Altered intestinal epithelial structure may impact the route and extent of absorption or passage through the intestinal lineage of exogenous as well as endogenous chemicals (74). A recent *in vitro* study also suggested basolateral exposure to low concentrations of DON to disrupt epithelial tight junction proteins and further barrier functionality of enteroids (103). This highlights the importance of investigations on cellular uptake and efflux of foodborne substances. Apical exposure to Uro A, AOH, and DON as single substances and in binary mixtures resulted in a significant passage of all three compounds (Figures 7A–C). The recovery of all three substances of interest or their metabolites within the cell lysate compartment, implies that the compounds can be taken up *via* the intracellular route in our experimental settings (104). Uro A and DON were mostly recovered unmodified (Uro A: 68 ± 9% – 129 ± 25%; DON: 93 ± 3% – 123 ± 1%), whereas AOH recovery rates were lower, ranging between 1.0 and 1.4% (Figure 7). Generally, the binary mixture exposure scenarios led to higher recovery rates (apical and basolateral compartment together) for all three substances. In relevance for the metabolism of Uro A, a recent Uro A intervention study described the bioavailability of DAP *in vivo*, with Uro A and its Phase II-metabolites reaching significant plasma levels within 24 h after oral supplementation (105). We found both, Uro A-S and Uro A-GlcA in all three compartments (Supplementary Figure 3). Nonetheless, high recoveries of the parent compound (Figure 7A) leave space for speculations concerning the kinetics of Uro A metabolism in our cell model. However, *in vivo* exposure to Uro A or structurally similar urolithins is considered to be substantially influenced by the individual metabolic competence, or “metabotype,” yielding different levels of formed urolithins depending on the persons’ age (106). In contrast, most recovered analytes concerning AOH, were observed as Phase II-metabolites, AOH-GlcA, AOH-3-S, and its monomethyl-ether AME (107). This is in line with the previous report of AOH to be rapidly absorbed, metabolized to AOH-GlcA and AOH-S, and transported to the basolateral side to a substantial degree within 3 h (108). Besides the distinct time frames investigated, a recent study suggests AOH to highly bind to human serum albumin (109): this aspect needs to be considered, when interpreting the overall low recoveries for all AOH analogs. Furthermore, AOH is also suggested to interact with cholesterol-rich cell membrane domains, possibly contributing to the reduced recoveries as well (40). As previously described, AOH is metabolized to AOH-S and AOH-GlcA in Caco-2 cells (110). Crudo et al. recovered Phase II-metabolites in a time-dependent manner, reaching the highest levels at 24 h of toxin exposure. Intriguingly, co-incubation with Uro C, a structural analog of Uro A, reduced the production of both AOH metabolites, and the distribution of AOH and the metabolites between the apical and basolateral compartment. Conversely, the exposure of Uro A to AOH led to no substantial alterations in metabolic or distribution pattern in our experimental setup (Figure 7 and Supplementary Figures 1–4). Nonetheless, the binary mixture of Uro A + AOH led to reduced recoveries of Uro A-GlcA, yet in turn, no significant change in AOH-GlcA recovery was detected (Supplementary Figures 3, 4). Overall, the co-exposure of DON to the two DAPs increased the recovery rates of not only the respective parent compounds, but also of Phase II-metabolites of Uro A (Uro-A-S in particular) and AOH (AOH-3-S, Supplementary Figure 2). By inhibiting the activity induction of Phase I-metabolism enzymes, CYP1A1/1A2/1B1 by Uro A and AOH (Figure 2), it is possible to hypothesize that more parent compounds are available as such for recovery (Figure 7) or for Phase II metabolism (Supplementary Figures 2, 3). Finally, DON was recovered mostly as the parent compound in our exposure scenario, showing almost equal distributions between the apical and the basolateral compartment. When DON was co-incubated with AOH, however, significantly more DON was found in the basolateral media compared to DON exposure alone (Figure 7). A recent study in Caco-2 cells reported DON to be metabolized to DON-3-S within 24 h; however, DON was applied in higher concentrations, and DON-3-S was recovered after 24 h in this case (67). Therefore, considering a possible production of DON-3-S in our cell model, concentrations would be too low to be considered for any impact on the endpoints measured in the study at hand.

Transcriptional activation of AhR target genes implies the regulation of several Phase I- and Phase II-metabolism enzymes, including CYP1A1, among others (18). After 6 h of substance incubation, 5 μM of B[a]P readily induced CYP1A1 relative gene transcription, while neither Uro A, AOH, nor the binary combination showed inductive potential (Supplementary Figure 18). On the contrary, the two DAPs significantly reduced mRNA transcription levels compared to the SC. The effect of Uro A and AOH toward the expression of CYP1A1 did not correlate with the EROD activities, suggesting that the expression of other CYP isoforms may be increased. Indeed, the outcome of the EROD assay measurements results from contributions of several CYP isoforms; thus, it cannot be excluded, that the individual isoforms might retain distinctive response profiles (111, 112). Accordingly, both DAPs could potentially also induce the enzyme activity of other CYP 1 family isoforms, such as CYP1A2/1B1, which are concomitantly addressed by EROD measurements as well (113, 114). Indeed, selectivity for the individual isoforms can occur in tissue- and substance-specific manners, as it was previously reported for B[a]P (115). In differentiated Caco-2, the effect of B[a]P on CYP1A1 (Figure 8 and Supplementary Figure 18) could be attributed to a higher sensitivity and/or different kinetic of the positive control when compared to Uro A and AOH. Of note, after 48 h of incubation, B[a]P induced EROD enzyme activity was of a much higher magnitude in comparison to that triggered by Uro A and AOH (Figures 2A–D). Other studies described enhanced mRNA transcription levels of CYP1A1 for both DAPs after 24 h of incubation, in different *in vitro* models (25, 116, 117). This contributes to the interpretation that Uro A and AOH could have different activity profiles in comparison to B[a]P in our cell model. The immunofluorescence data (Figure 8) could support this perception. The CYP1A1 signal in cells incubated with the binary mixture of Uro A + AOH was significantly higher in comparison to AOH alone, a response which infers for the capability of the substances to affect this pathway, albeit with a different threshold of activation.

In sum, the sub-cytotoxic concentrations (Figure 3) of Uro A and AOH applied to the differentiated Caco-2 monolayer, yielded additive efficacy toward EROD enzyme activity (Figure 2), and monolayer integrity measured as TEER enhancements (Figure 4). While AOH rather reduced both ZO-1 as well as CYP1A1 immunofluorescence signal, Uro A partially antagonized these effects (Figures 6, 8). The activity profile of DON seemed centered on other molecular levers and dominated by the proteostatic effect. Based on the structural similarity of AOH and Uro A, it seems legitimate to postulate alike mechanisms of action (41). However, AOH is classified as mycotoxin, while Uro A is primarily described for its health benefits (118), as it is considered safe for use as a dietary supplement (119). In any case, supplementation of ellagitannins containing fruit extracts, in combination with the respective “metabotype” account for considerable exposure of the intestinal epithelium. Of note, urolithin precursors were reported for their potential to inhibit topoisomerase II catalytically at low concentrations, and although low concentrated Uro A and Uro B could not sustain this effect, knowledge on the impact of higher concentrations in this respect is lacking (120). Moreover, a few studies suggested Uro A to interfere with hormone-dependent signaling (121, 122), which is plausible, since DAPs structurally resemble endogenous hormones, such as 17-β-oestradiol (E2) for example. On this note, the highly complex AhR signaling is regularly reported to interfere with physiological and pathological processes on multiple levels, including hormone metabolism and hormone-receptor-signaling (123). Cross-talks with other transcription factors and downstream signaling cascades can yield varying outcomes, necessitating further assessment of substances interacting with the signaling of AhR and its subsequent effects on human health. These are suspected to go way beyond the metabolism of environmental pollutants to potential carcinogens, and affect the immune responses, hormonal cascades, and the epithelial integrity of barrier tissues, such as the colon (41, 84, 124, 125).

## Concluding Remarks

Taken together, this study reports for the first time, single and combinatory effects of Uro A, AOH, and DON on intestinal AhR activation and implications toward the epithelial structural integrity and metabolic capacities of colon cancer. CYP1A1/1A2/1B1 enzyme activating potential of Uro A and AOH was diminished by co-exposure to DON, an effect reversed by exchanging DON for its human metabolite DON-3-S. Combination of the two food-derived DAPs showed to rather support the colonic barrier integrity. However, AOH did not pair the activity of Uro A in the modulation of CYP1A1 and ZO-1 localization, suggesting that the molecular pathways of the two compounds must separate downstream the AhR pathway activation. Conversely, DON altered the intestinal structure and efflux of Uro A, AOH, and their metabolites to the apical or basolateral compartment, respectively. Thus, considering the human colonic tissue being regularly exposed to chemical mixtures, more in-depth investigations on foodborne small molecules and their interactive impact on intestinal health and absorptive/metabolic capacities is required to support the risk assessment and ensure food safety.

## Data Availability Statement

The original contributions presented in this study are included in the article/Supplementary Material, further inquiries can be directed to the corresponding authors.

## Author Contributions

JG, GDF, and DM: conceptualization. JG and DM: data curation and project administration. JG and CS: formal analysis. JG: investigation, visualization, and writing—original draft. JG, GDF, EV, and DM: methodology. DM and GDF: resources. EV, GDF, and DM: supervision. JG, CS, EV, GDF, and DM: validation. EV, CS, GDF, and DM: writing, review and editing. All authors have read and agreed to the published version of the manuscript.

## Conflict of Interest

The authors declare that the research was conducted in the absence of any commercial or financial relationships that could be construed as a potential conflict of interest.

## Publisher’s Note

All claims expressed in this article are solely those of the authors and do not necessarily represent those of their affiliated organizations, or those of the publisher, the editors and the reviewers. Any product that may be evaluated in this article, or claim that may be made by its manufacturer, is not guaranteed or endorsed by the publisher.
