# Supplementary material for: Combinatory Exposure to Urolithin A, Alternariol, and Deoxynivalenol Affects Colon Cancer Metabolism and Epithelial Barrier Integrity in vitro
Source: Front Nutr. 2022 Jun 24;9:882222. doi: 10.3389/fnut.2022.882222 (PMC9263571; doi:10.3389/fnut.2022.882222)
Supplement: Supplementary file 1 [file Data_Sheet_1.PDF]

Supplementary Material

1 Supplementary Figures

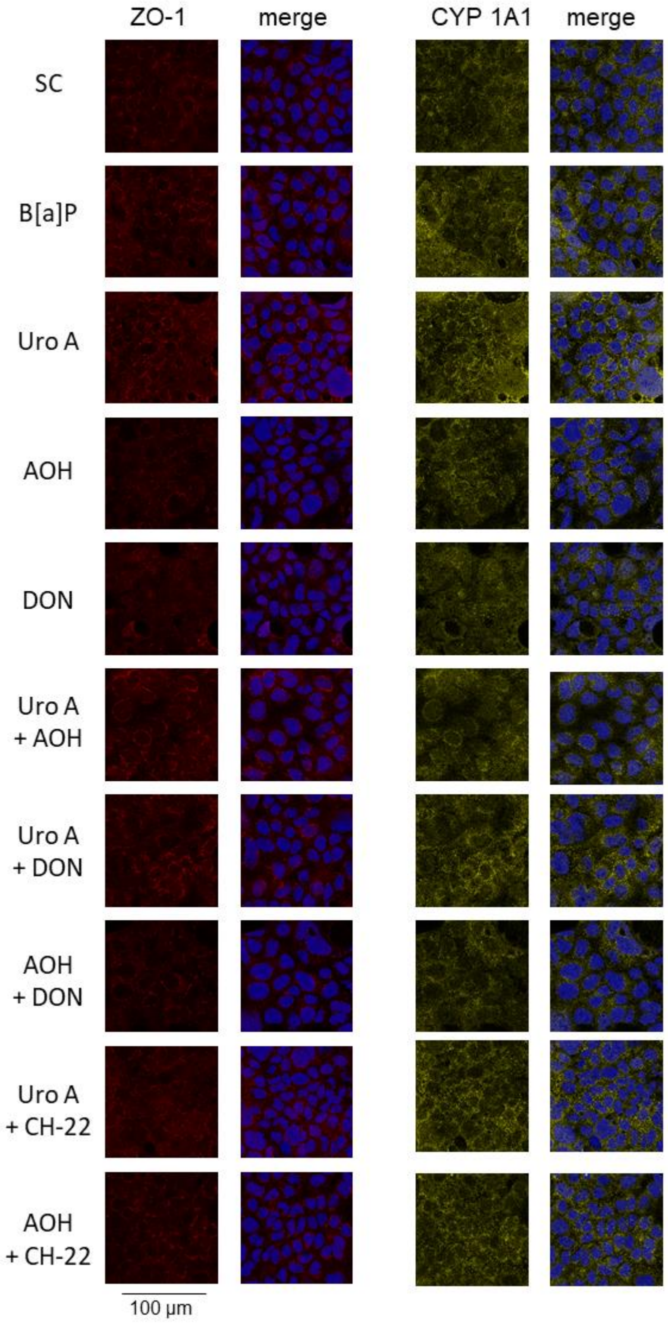

Supplementary Figure 1. Immunofluorescence staining of zona occludens-1 (ZO-1) and Cytochrome P 450 1A1 (CYP1A1) protein after 48 hours of incubation. The panel is supplemented with complement incubation conditions, which do not occur in the main manuscript, or are missing in Supplementary Figure 1 (+ CH22). Images obtained from a 710 LSM Zeiss microscope (63X objective). See manuscript for detailed description of staining and imaging method.

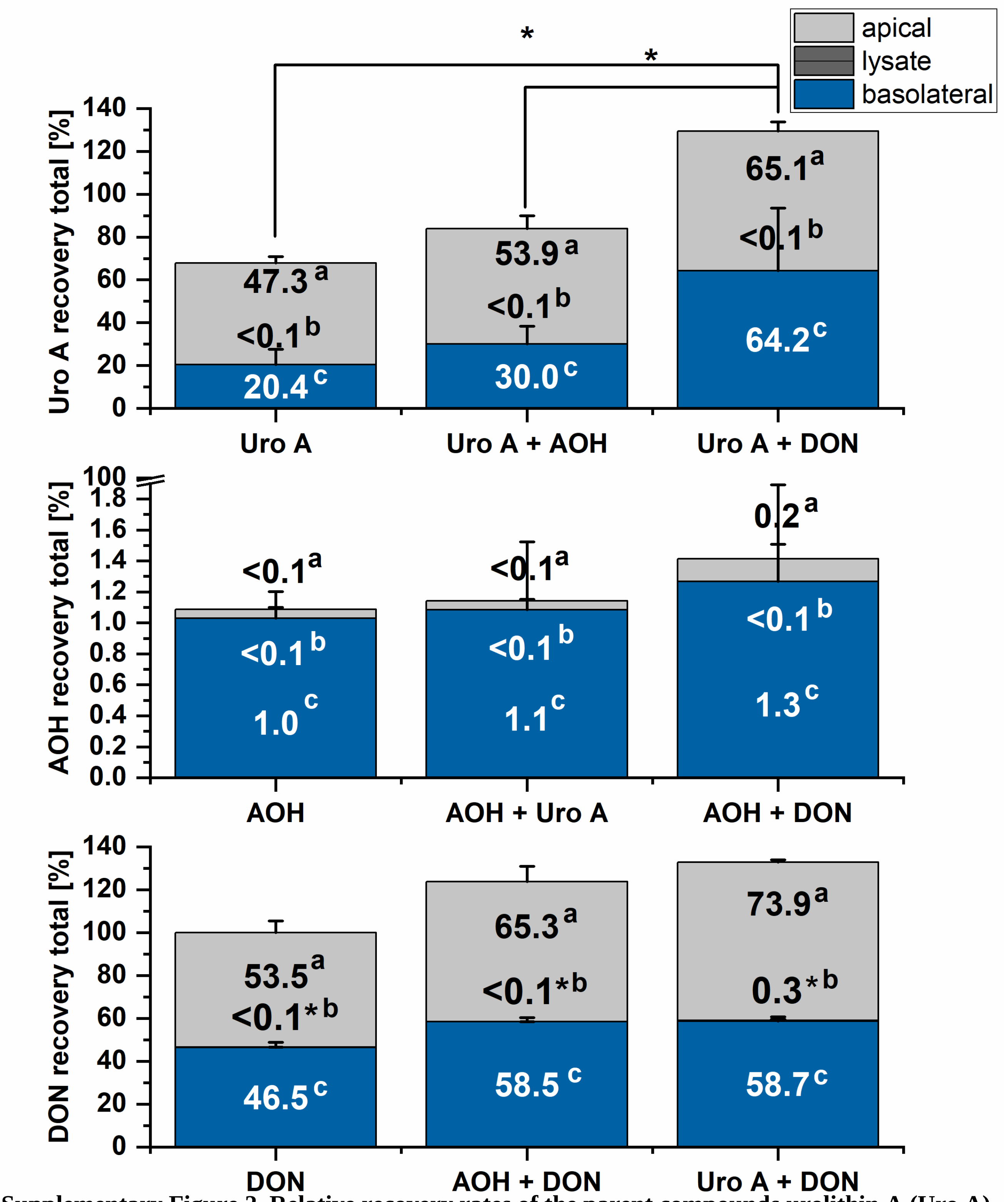

Supplementary Figure 2. Relative recovery rates of the parent compounds urolithin A (Uro A), alternariol (AOH), and deoxynivalenol (DON) in the respective compartment: (a) apical, (b) lysate, or (c) basolateral. Sum of area under the curve (AUC) of Uro A incubation conditions was set to 100 %. * mark conditions, for which <3 biological replicates were suitable for analysis of the compound of interest. For more details see manuscript Sections 2.8, 3.7, and 4.6.

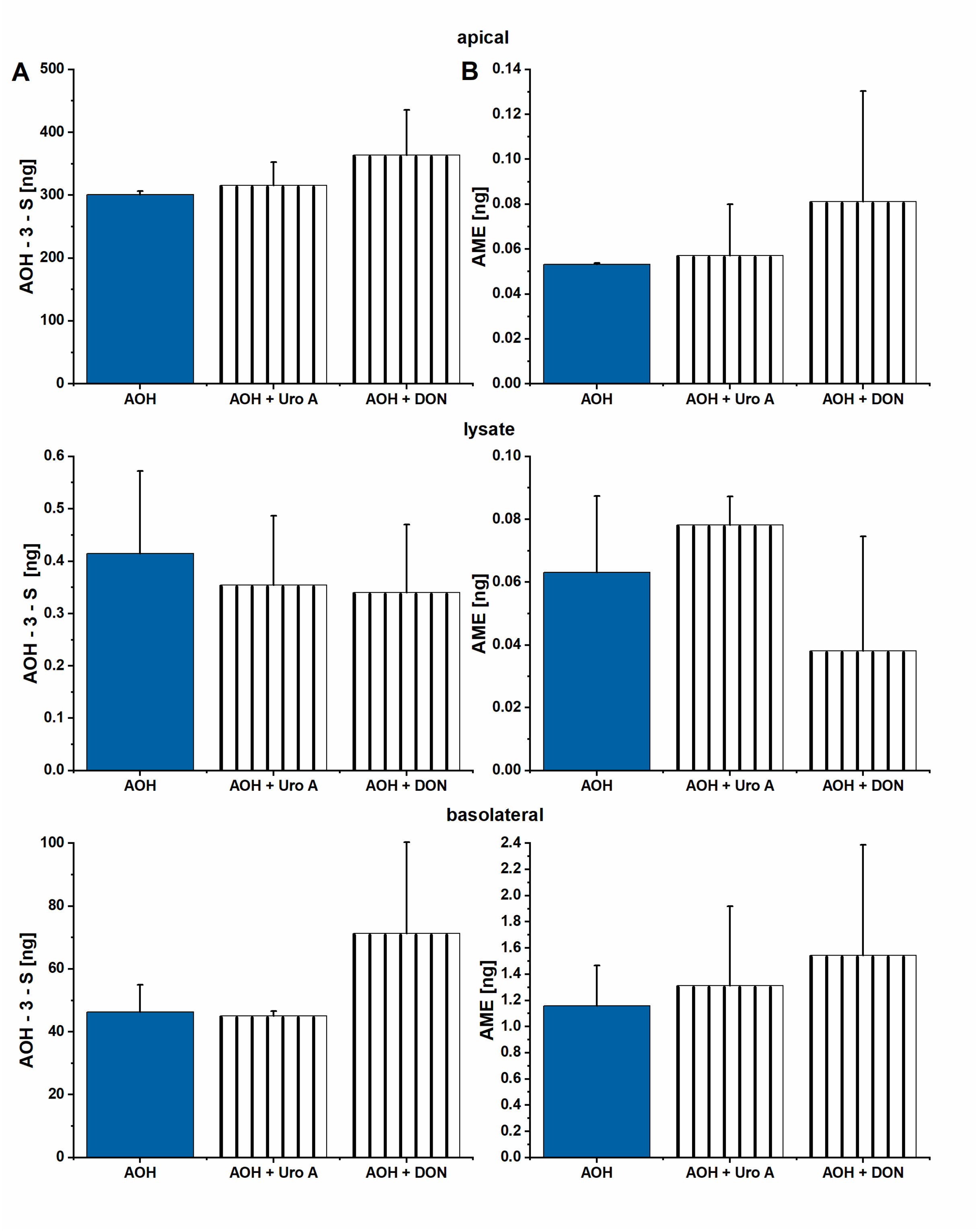

Supplementary Figure 3. Recoveries (ng) of alternariol-3-sulfate (AOH-3-S) and alternariol monomethyl ether (AME) in the apical, lysate, and basolateral media.

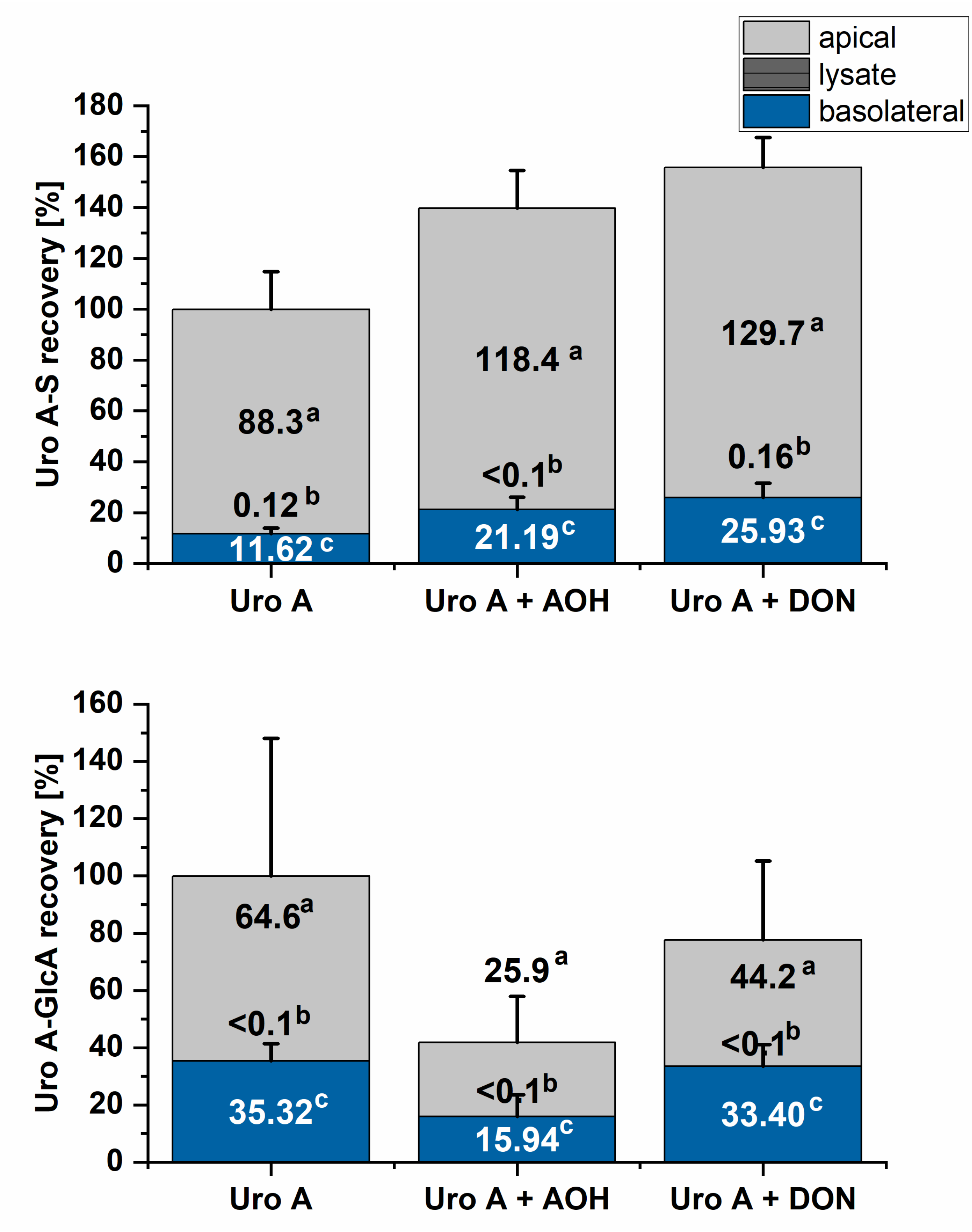

Supplementary Figure 4. Relative recoveries of Uro A metabolites: Uro A-S and Uro A-GlcA in the respective compartments: (a) apical, (b) lysate, and (c) basolateral. Sum of area under the curve (AUC) of Uro A incubation conditions was set to 100 %. Other shares in the three compartments and recoveries in other incubation conditions were related to Uro A.

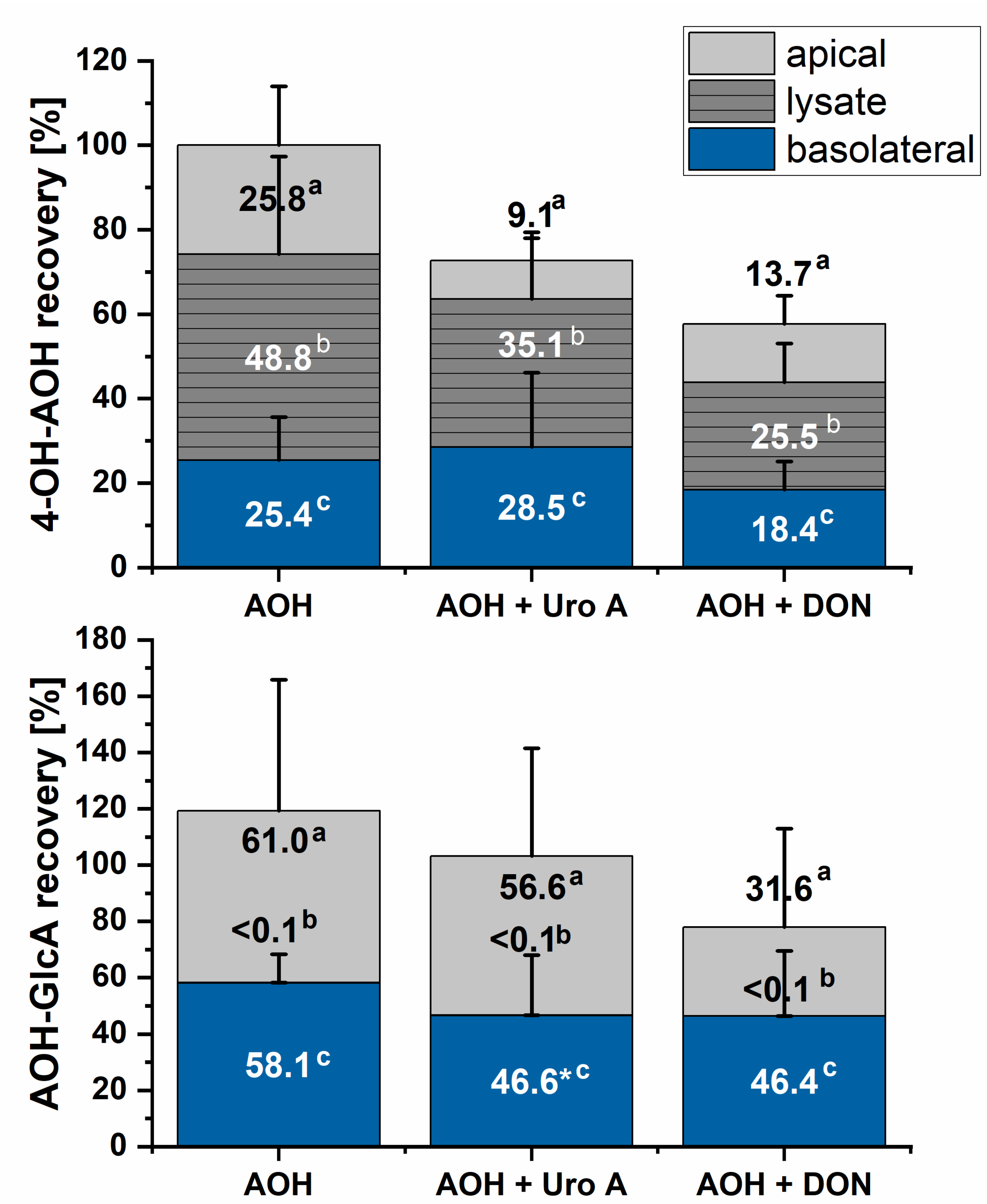

Supplementary Figure 5. Relative recoveries of AOH metabolites: 4-OH-AOH and AOH-GlcA in the respective compartments: (a) apical, (b) lysate, and (c) basolateral. Sum of area under the curve (AUC) of AOH incubation conditions was set to 100 %. Other shares in the three compartments and recoveries in other incubation conditions were related to AOH. * mark conditions, for which <3 biological replicates were suitable for analysis of the compound of interest.

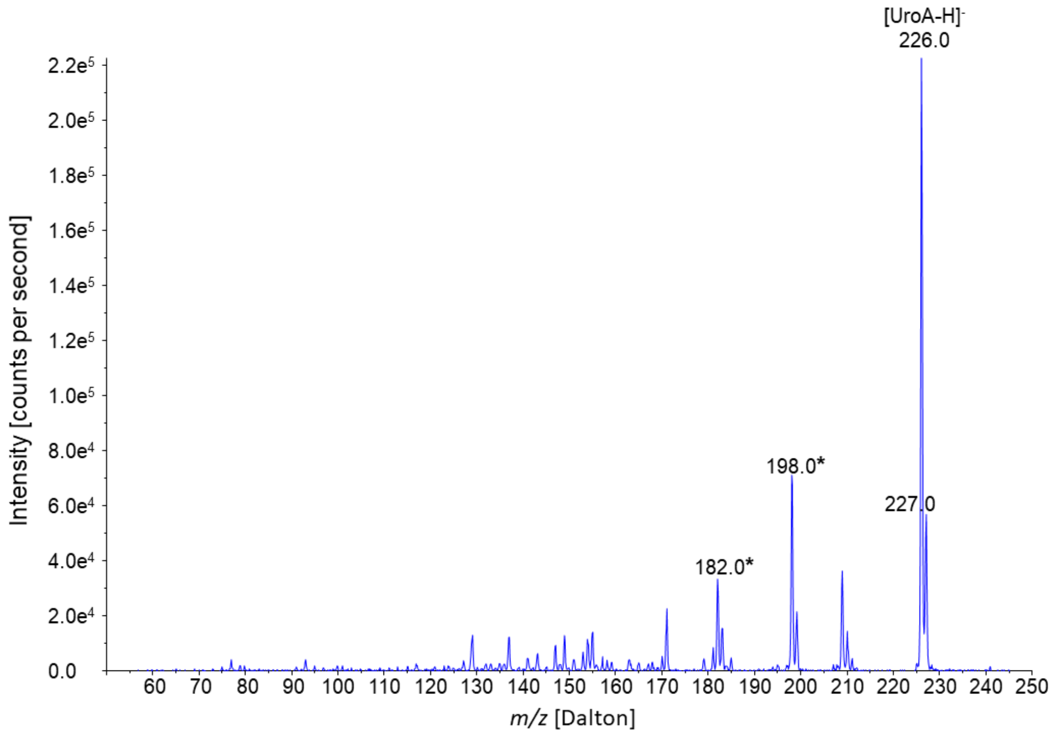

Supplementary Figure 6. Enhanced product ion (EPI) scan of urolithin A (Uro A). The precursor is annotated, and the respective mass transitions are marked with *. The scan was acquired at a collision energy (CE) of -40 V.

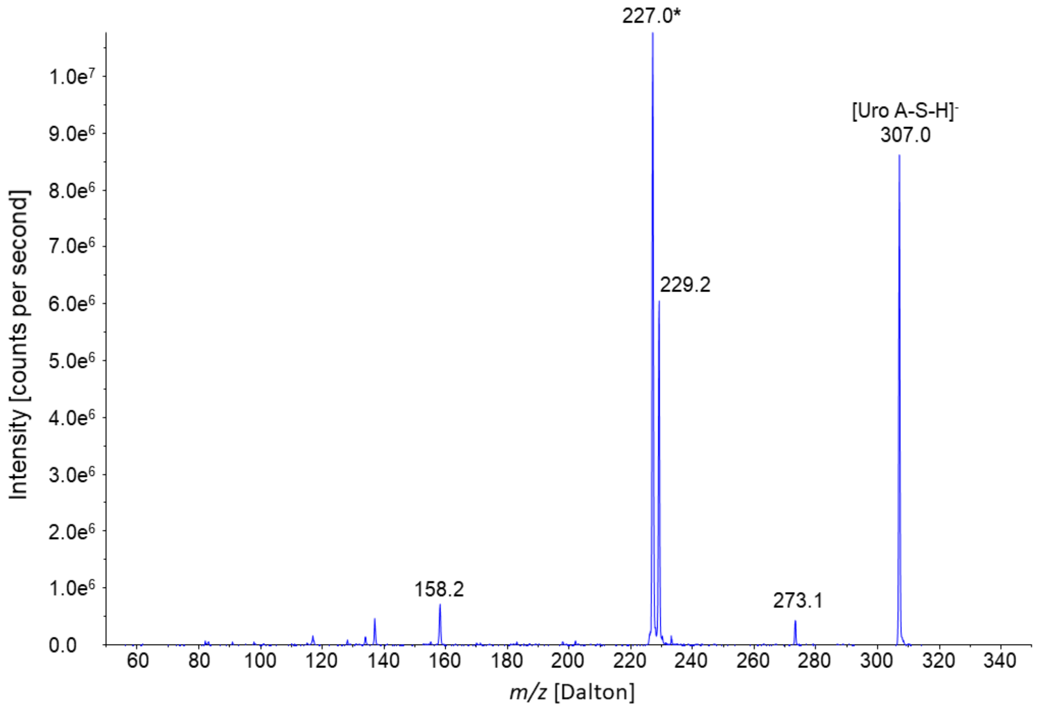

Supplementary Figure 7. EPI scan of urolithin A-sulfate (Uro A-S). The precursor is annotated, and the respective mass transitions are marked with *. The scan was acquired at a CE of -20 V.

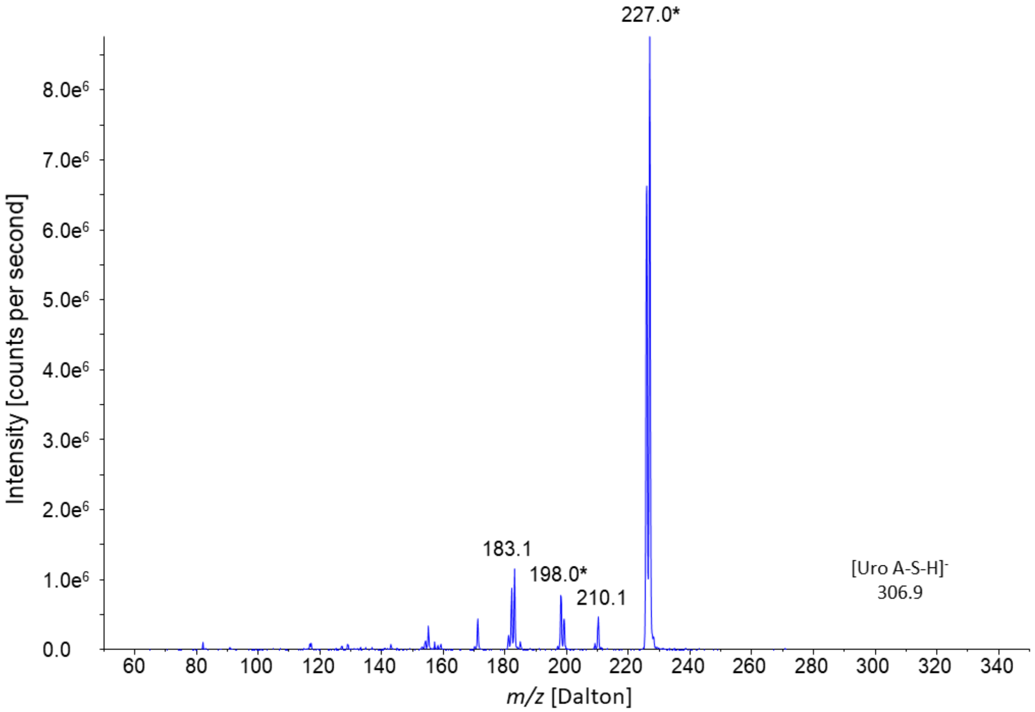

Supplementary Figure 8. EPI scan of urolithin A-sulfate (Uro A-S). The precursor is annotated, and the respective mass transitions are marked with *. The scan was acquired at a CE of -50 V.

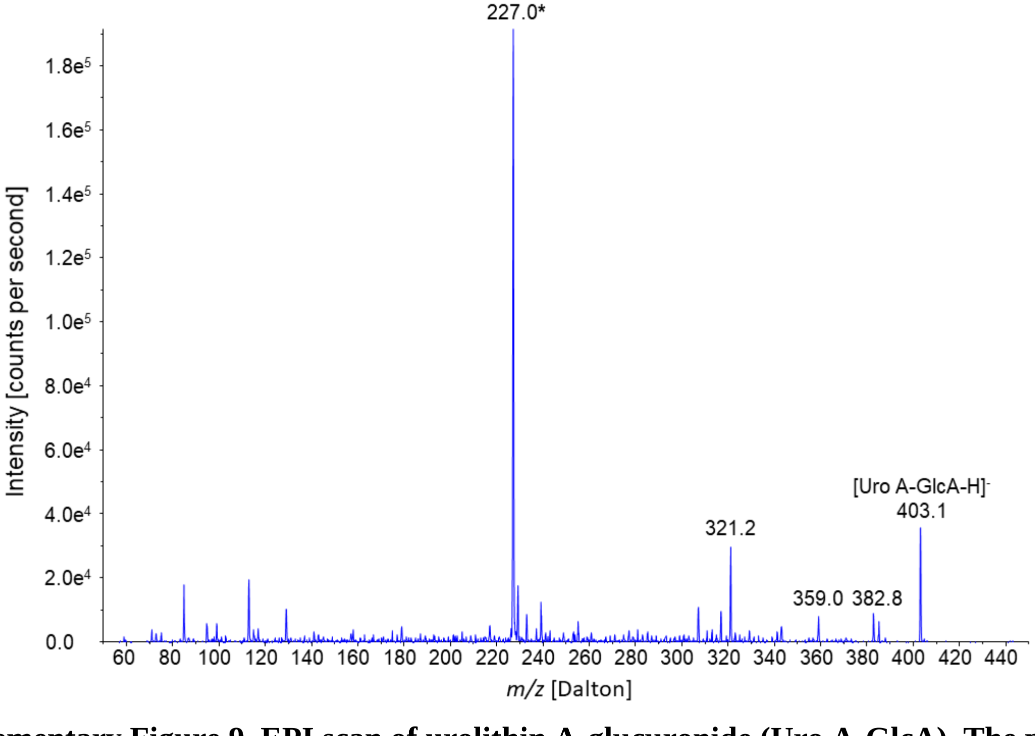

Supplementary Figure 9. EPI scan of urolithin A-glucuronide (Uro A-GlcA). The precursor is annotated, and the respective mass transitions are marked with *. The scan was acquired at a CE of -30 V.

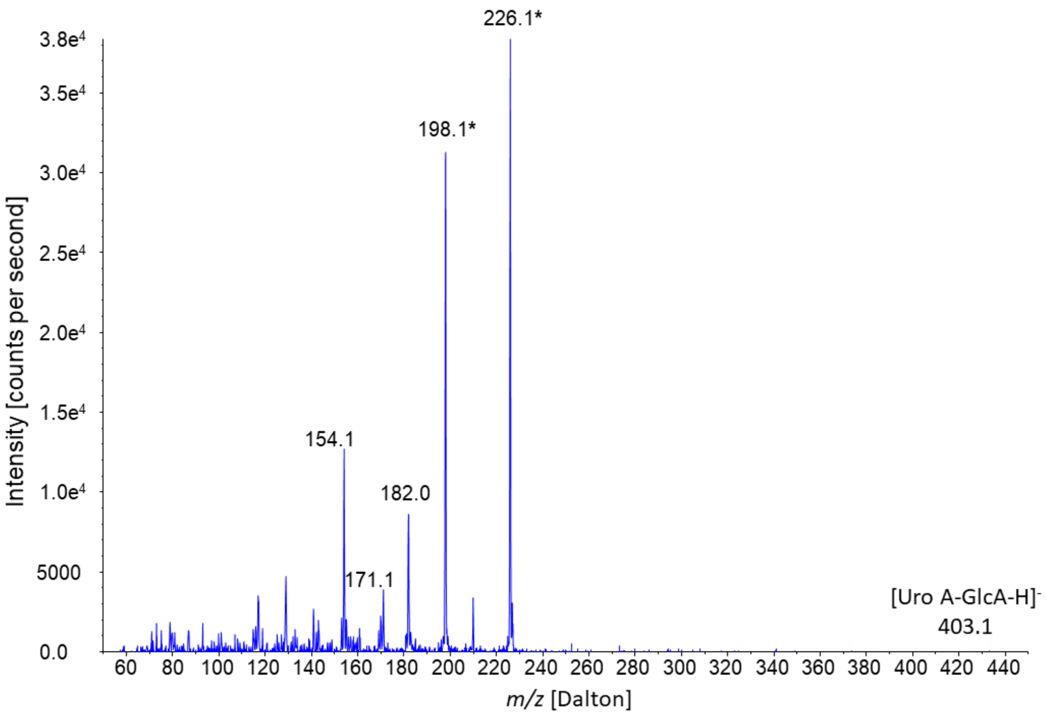

Supplementary Figure 10. EPI scan of urolithin A-glucuronide (Uro A-GlcA). The precursor is annotated, and the respective mass transitions are marked with *. The scan was acquired at a CE of -90 V.

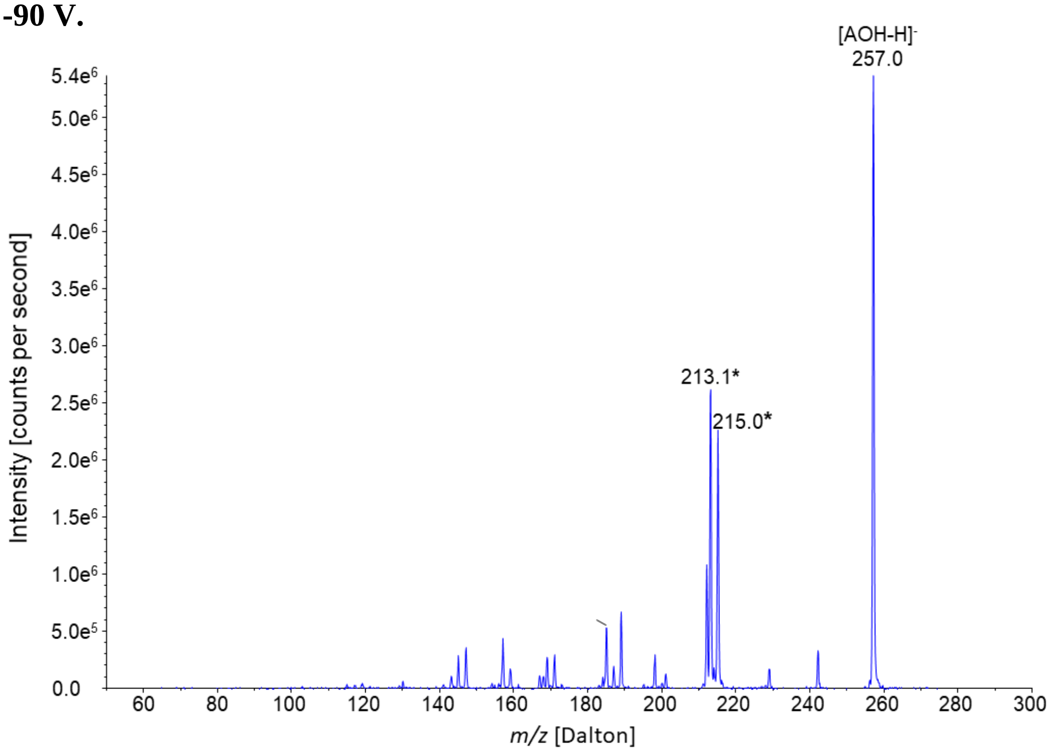

Supplementary Figure 11. EPI scan of alternariol (AOH). The precursor is annotated, and the respective mass transitions are marked with *. The scan was acquired at a CE of -35 V.

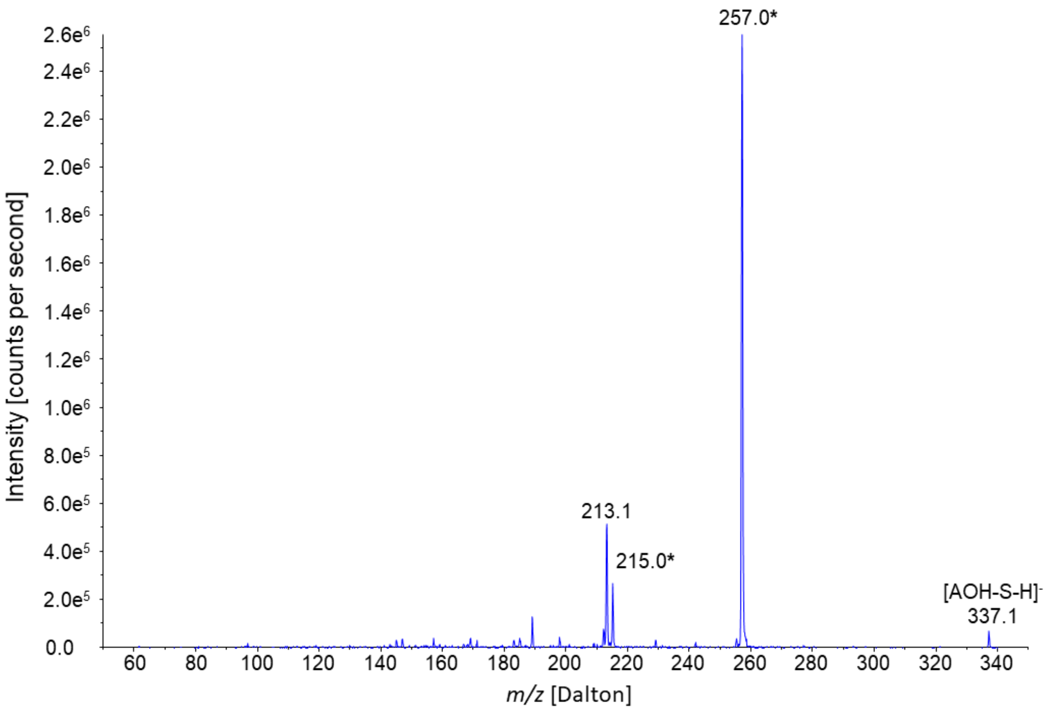

Supplementary Figure 12. EPI scan of alternariol-sulfate (AOH-S). The precursor is annotated, and the respective mass transitions are marked with *. The scan was acquired at a CE of -45 V.

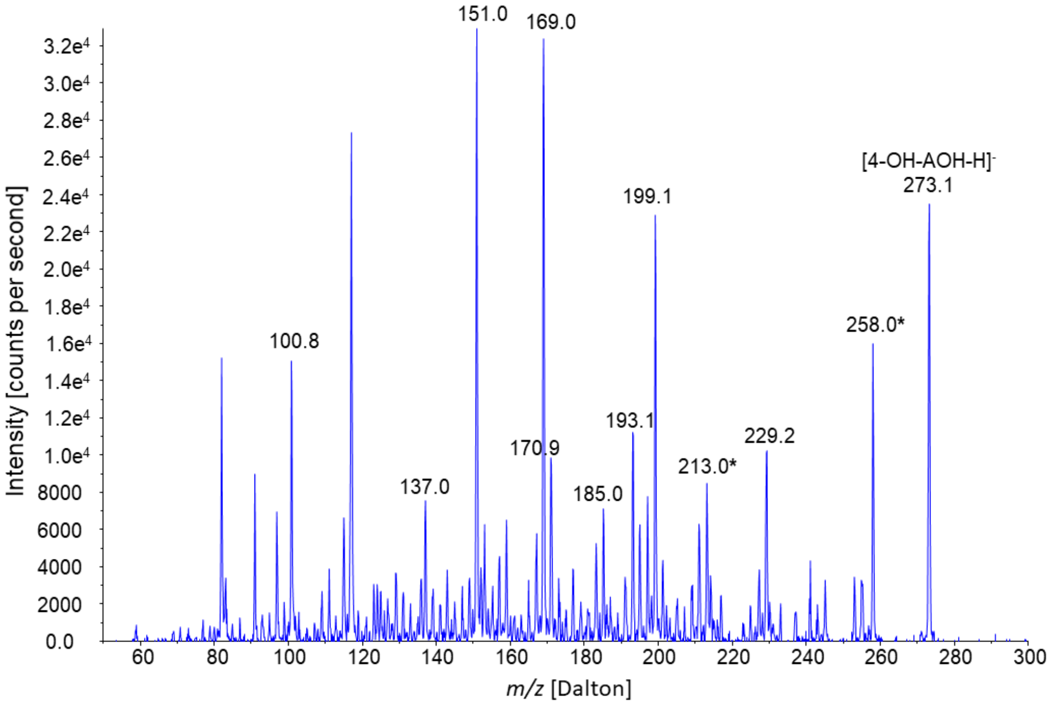

Supplementary Figure 13. EPI scan of 4-hydroxy-alternariol (4-OH-AOH). The precursor is annotated, and the respective mass transitions are marked with *. The scan was acquired at a CE of -35 V.

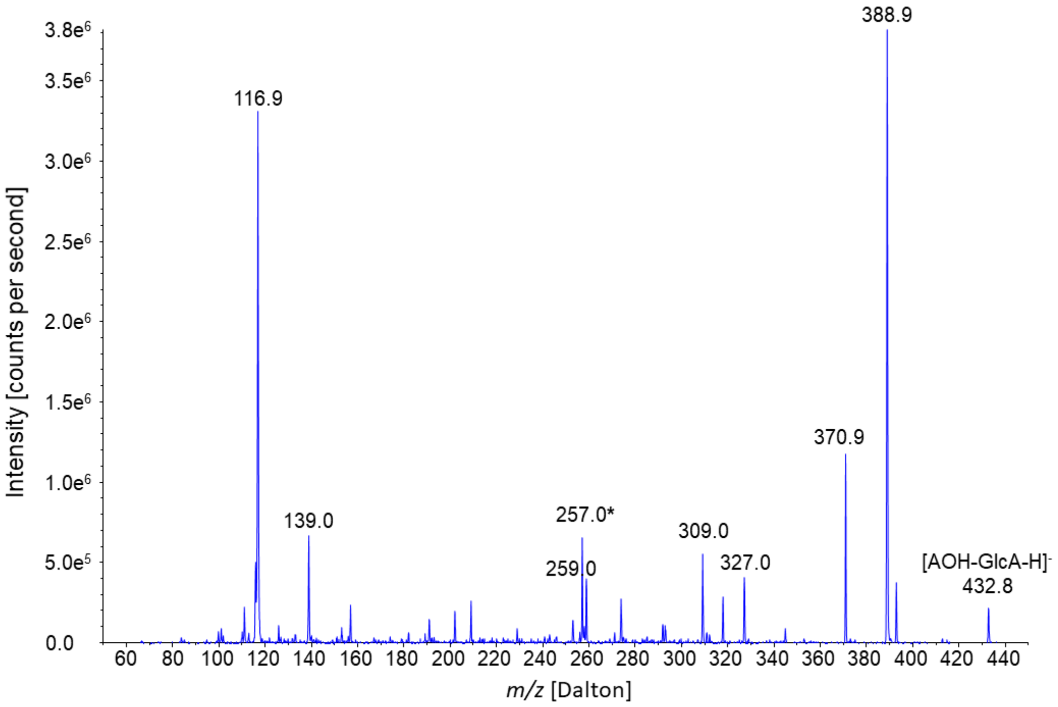

Supplementary Figure 14. EPI scan of alternariol-glucuronide (AOH-GlcA). The precursor is annotated, and the respective mass transitions are marked with *. The scan was acquired at a CE of -25 V.

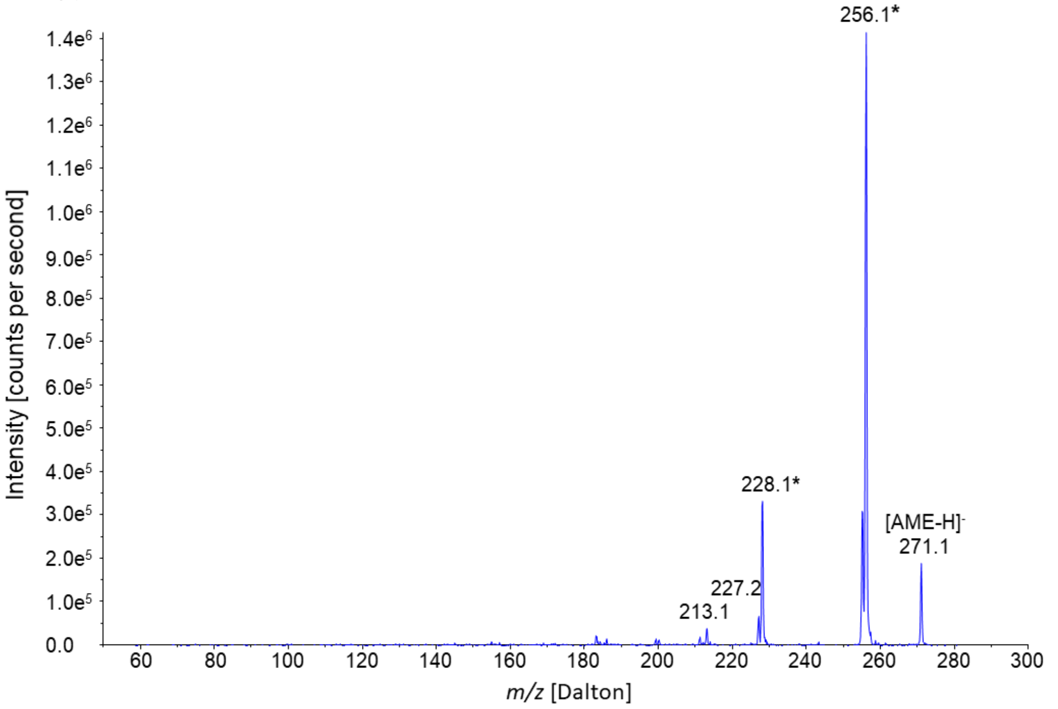

Supplementary Figure 15. EPI scan of alternariol monomethyl ether (AME). The precursor is annotated, and the respective mass transitions are marked with *. The scan was acquired at a CE of -35 V.

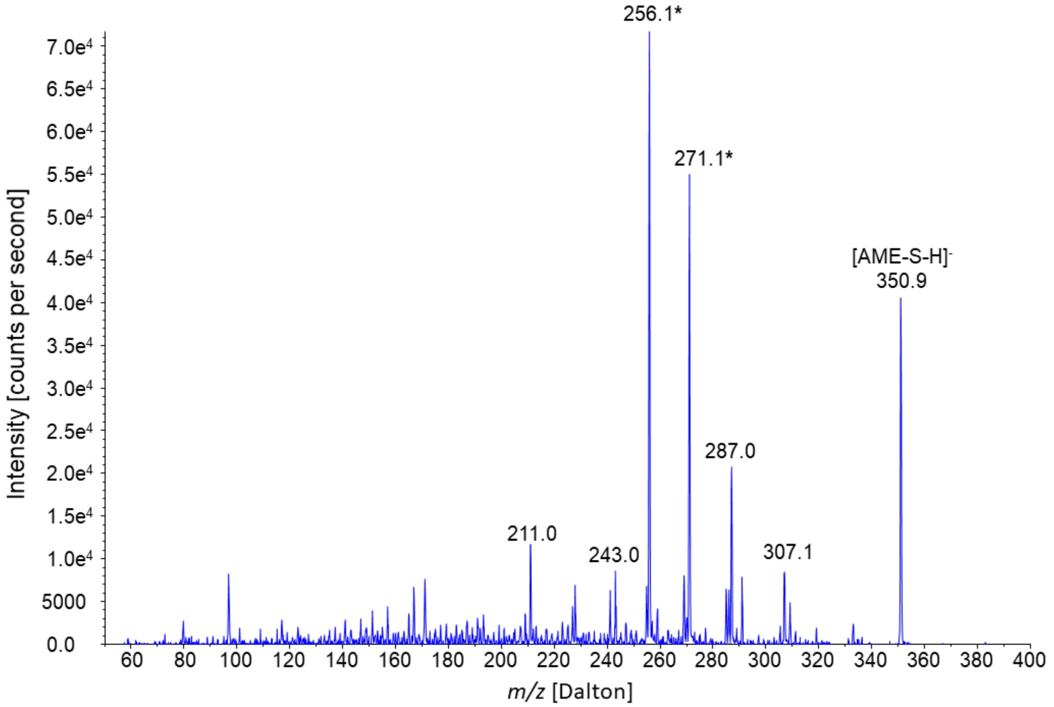

Supplementary Figure 16. EPI scan of alternariol monomethyl ether-sulfate (AME-S). The precursor is annotated, and the respective mass transitions are marked with *. The scan was acquired at a CE of -45 V.

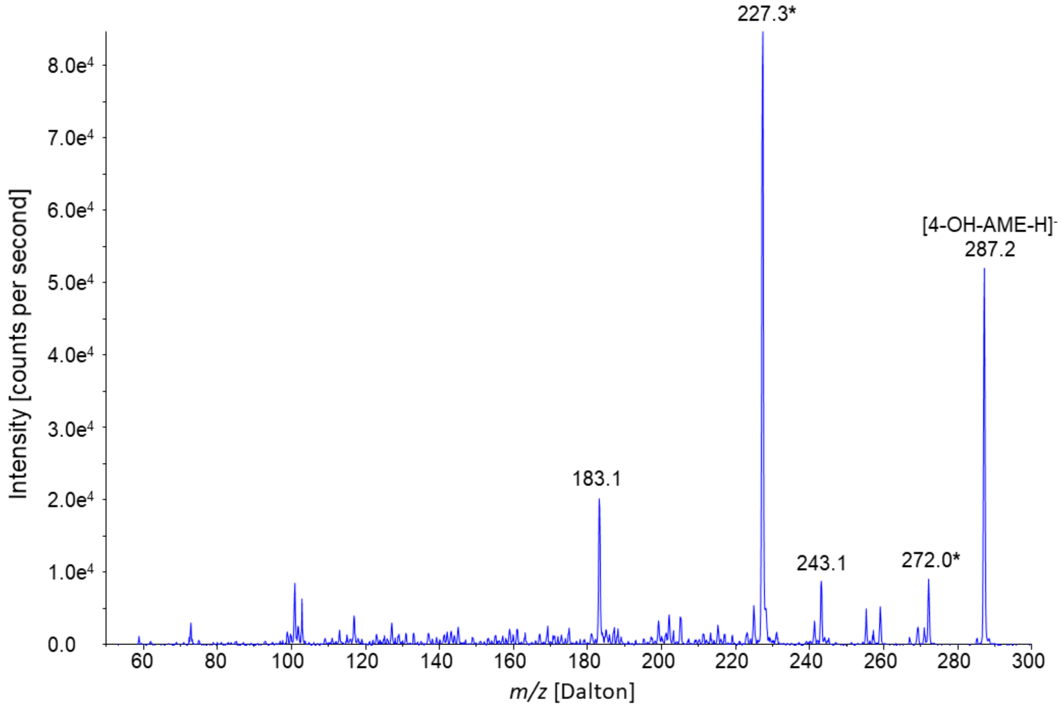

Supplementary Figure 17. EPI scan of 4-hydroxy-alternariol monomethyl ether (4-OH-AME). The precursor is annotated, and the respective mass transitions are marked with *. The scan was acquired at a CE of -30 V.

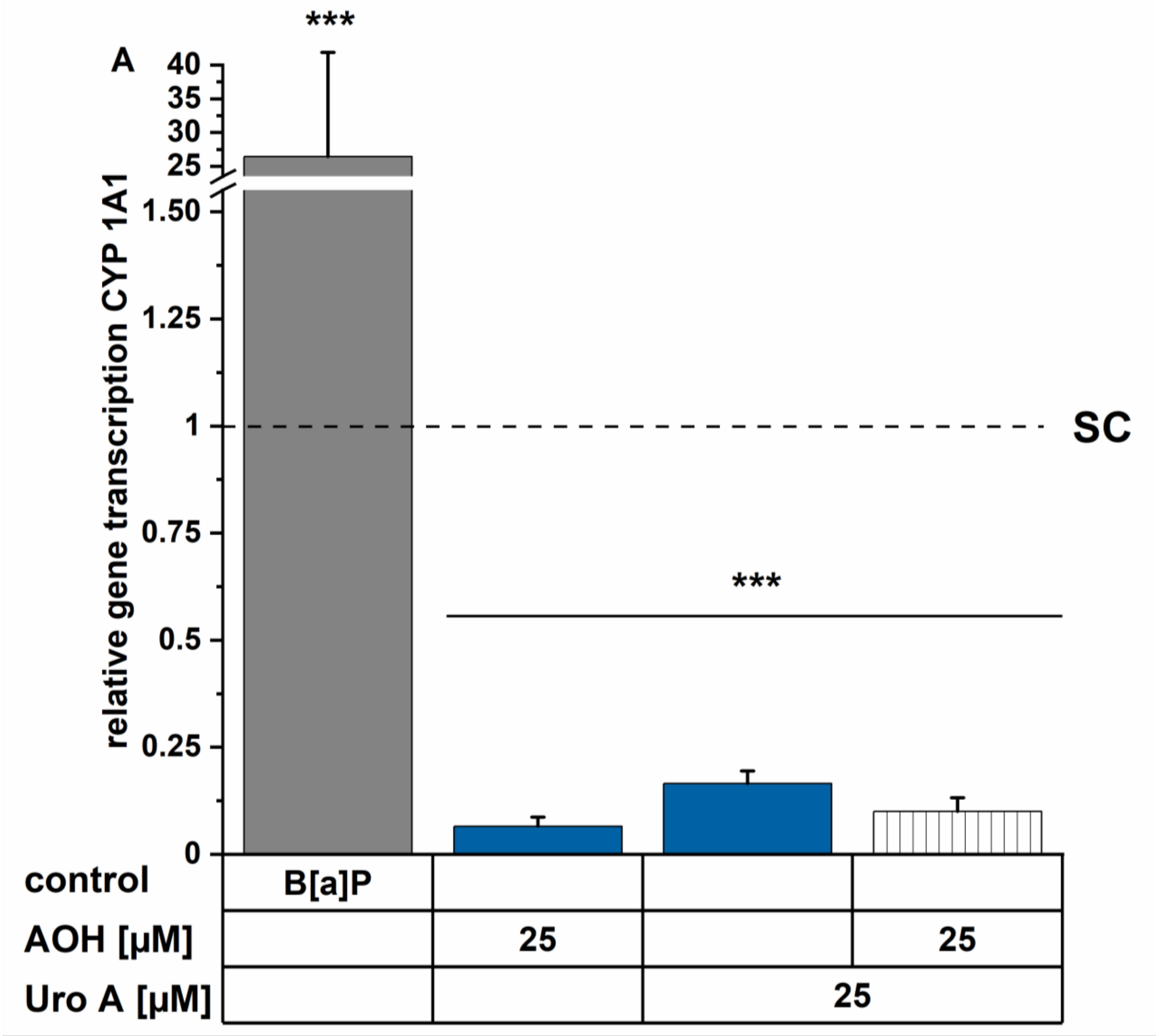

Supplementary Figure 18. (A) q-RT PCR experiments. Relative gene transcription levels after 6 hours of substance exposure. * (*p<0.001*) indicates significant differences compared to the solvent control, calculated using two-samples Students' *t*-test. (See manuscript Section 3.8 (Materials & Methods) for detailed description of q-RT PCR experiments. Results are described in Sections 4.7 (Results).**

Experiment	Outcomes & interactions					
EROD	↑	↑↑	↔	↑↑	↔	↔
EROD + IL-1β	↑	↑↑	↔	↑↑	↔	↔
Neutral Red	↔	↔	↓	↔	↔	↓
NR + IL-1β	↔	↔	↓	↔	↓	↓
TEER 48 hrs	↑	↑	↓	↑↑	↔	↔
Lucifer Yellow	↔	↑	↔	↑	↑	↑
ZO-1 protein	↔	↓	↓	↑↑	↑	↑
Recovery Uro A	↔			↑	↑↑	
Recovery AOH		↔		↔		↑
Recovery DON			↔		↑	↑
qRT-PCR CYP1A1	↓	↓		↓		
CYP1A1 protein	↔	↓	↓	↔	↔	↔
**Uro A**	**X**			**X**	X	
**AOH**		**X**		**X**		X
**DON**			X		X	X

Supplementary Table 1. Overview of single exposure effects vs. combinatory impact on the respective experiments. Uro A and AOH are highlighted for more clarity on how the two dibenzo-α-pyrones interact in additive/antagonistic tendencies towards each other.
